# Bidirectional control of vesicular GABA release, LTP, and amotivation by GluN2D-selective allosteric modulators and ketamine

**DOI:** 10.1016/j.celrep.2026.117707

**Published:** 2026-07-24

**Authors:** Viktor J. Oláh, Isabelle F. Witteveen, Tue G. Banke, Alexa M. Leahy, Ryan Keable, Riley E. Perszyk, Cara T. Khayat, TrangKimberly T. Nguyen, Brianna J. Bixler, Frances G. Ghinger, Detlef Vullhorst, Yeeun Yook, Scott J. Myers, Elijah Z. Ullman, Jing Zhang, Hao Xing, John F. Traynelis, Eva S. Diaz, Sukhan Kim, Sophia Boraschi, Nicholas S. Akins, Ken H. Liu, Alet van der Westhuyzen, Yanli Yang, Chad R. Camp, Andres Buonanno, Randy A. Hall, James Q. Zheng, Dennis C. Liotta, Matthew J. Kennedy, Katherine W. Roche, Shannon L. Gourley, Hongjie Yuan, Matthew J.M. Rowan, Stephen F. Traynelis

**Affiliations:** 1 Department of Cell Biology, Emory University School of Medicine, Atlanta, GA 30322, USA; 2 Department of Pharmacology and Chemical Biology, Emory University School of Medicine, Atlanta, GA 30322, USA; 3 Department of Pediatrics, Emory University School of Medicine, Atlanta GA 30322, USA; 4 National Institute of Neurological Disorders and Stroke, Bethesda, MD 20892, USA; 5 National Institute of Child Health and Human Development, Bethesda, MD 20892, USA; 6 Department of Pharmacology, University of Colorado-Denver, Aurora CO 80045, USA; 7 Department of Chemistry, Emory University, Atlanta GA 30322, USA; 8 Center for Neurodegenerative Disease, Emory University School of Medicine, Atlanta, GA 30322, USA; 9 Senior author; 10 These authors contributed equally; 11 Present address: Department of Cellular and Molecular Medicine, Florida International University, Miami, FL 33199, USA; 12 Lead contact

## Abstract

GABAergic inhibitory interneurons balance brain activity by suppressing excessive excitatory signaling and shaping network oscillations. Despite this critical function, our understanding of how neural circuits regulate GABAergic systems remains limited. Although N-methyl-D-aspartate receptor (NMDAR) functionality is defined by the GluN2A-D subunits, the interneuron NMDAR subunit composition remains unknown. Here, we show that GluN2D-NMDARs reside at presynaptic terminals of hippocampal parvalbumin-interneurons, and that the GluN2D intracellular C-terminal contains presynaptic targeting information. GluN2D-NMDARs are activated by changes in basal glutamate to bidirectionally control GABA release. Importantly, inhibition of GABA release by GluN2C/D modulators and subanesthetic ketamine is absent in mice lacking GluN2D in parvalbumin-interneurons, suggesting that GluN2D-dependent presynaptic regulation of GABA release may underlie some of ketamine’s effects. The GluN2C/D-selective inhibitor NAB14 recapitulates durable ketamine-induced enhancement of long-term potentiation (LTP) and recovery of reward motivation in a behavioral model of depression without ketamine’s undesirable effects, suggesting that GluN2D-NMDARs could be a therapeutically relevant target.

## In brief

Olah et al. show that GluN2D is a presynaptic regulator of GABA release in hippocampal parvalbumin-interneurons. Ketamine and GluN2C/D-selective modulation of GABA release and LTP are absent in mice lacking GluN2D in parvalbumin-interneurons. We show that both ketamine and GluN2C/D inhibition ameliorate amotivation in a behavioral model of depression.

## Graphical abstract

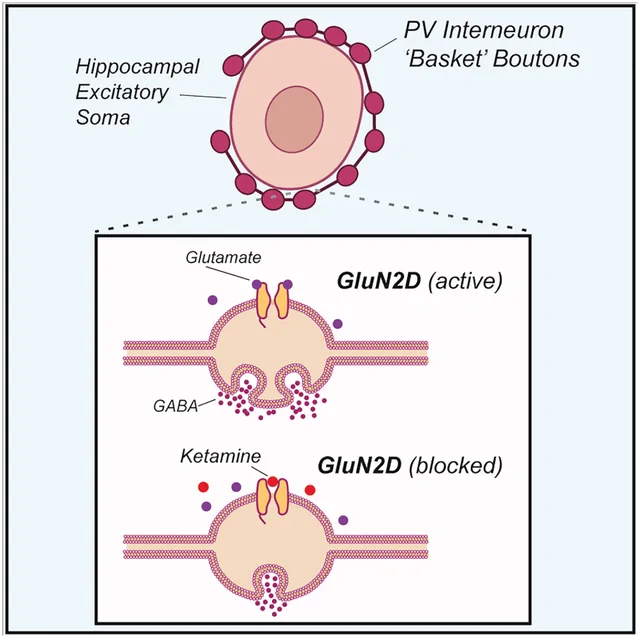

## INTRODUCTION

Three types of glutamate receptors (α-amino-3-hydroxy-5-methyl-4-isoxazolepropionic acid (AMPA), kainate, and N-methyl-D-aspartate receptors (NMDARs)) mediate excitatory synaptic transmission in the central nervous system. Of these, NMDARs have garnered considerable interest for their roles in learning, memory, development, and neuropsychiatric disorders.^1,2^ NMDARs are tetrameric assemblies that contain GluN1 and any of four GluN2 subunits (GluN2A-D), and are activated by the co-agonists glutamate and glycine.^1^ NMDARs that contain the GluN2D subunit display dramatically different functional properties than other GluN2 subunits, including exceptionally low open probability (~0.02), nanomolar glutamate potency, slow deactivation time course, and no desensitization.^1^ These characteristics are inconsistent with those of typical postsynaptic receptors. Moreover, GluN2D is expressed at lower levels in the hippocampus and cortex than GluN2A or GluN2B,^3^ both of which are postsynaptic receptors. Inhibitory interneurons, which are critical for maintaining normal brain activity and preventing aberrant synaptic plasticity,^4,5^ strongly express GluN2D.^2,6–8^ Whereas these observations suggest a unique role for GluN2D in circuit function, there is a paucity of data on GluN2D due to both the lack of reliable antibodies and (until recently) selective pharmacological tools. While a great deal is known about GluN2A-C, the lack of information about GluN2D raises an important question—what role does GluN2D serve in the healthy brain and in complex brain disorders?

Several hints have emerged in terms of roles that GluN2D-containing receptors may play, given its expression pattern in the cortex and hippocampus.^6–8^ For example, the GluN2C/D-selective positive allosteric modulators (PAMs) increase both the amplitude of NMDAR-mediated EPSCs in hippocampal CA1 interneurons^8,9^ and the inhibitory component of compound postsynaptic excitatory-inhibitory potentials in response to Schaffer collateral stimulation.^9^ Field potential recordings of voltage fluctuations in hippocampal slices reveal that potentiation of GluN2C/D-NMDARs can increase gamma oscillations (30–100 Hz^9^), reflecting the modulation of fast-spiking PV-interneuron activity.^10^ However, these data do not distinguish between postsynaptic or presynaptic roles of GluN2D-NMDARs. To address this question, we engineered a mouse to visualize extracellular GluN2D and combined super-resolution imaging, electrophysiological recordings, and behavioral pharmacology to show that the unexpected expression of glutamate/glycine-activated GluN2D-NMDARs on presynaptic terminals of inhibitory PV-interneurons mediates some of the actions of subanesthetic ketamine. Thus, GluN2D inhibition may be a potential therapeutic strategy for the treatment of depression.

## RESULTS

### GluN2D-SEP signals are enriched in PV-interneuron basket synapses

**Figure 1.**
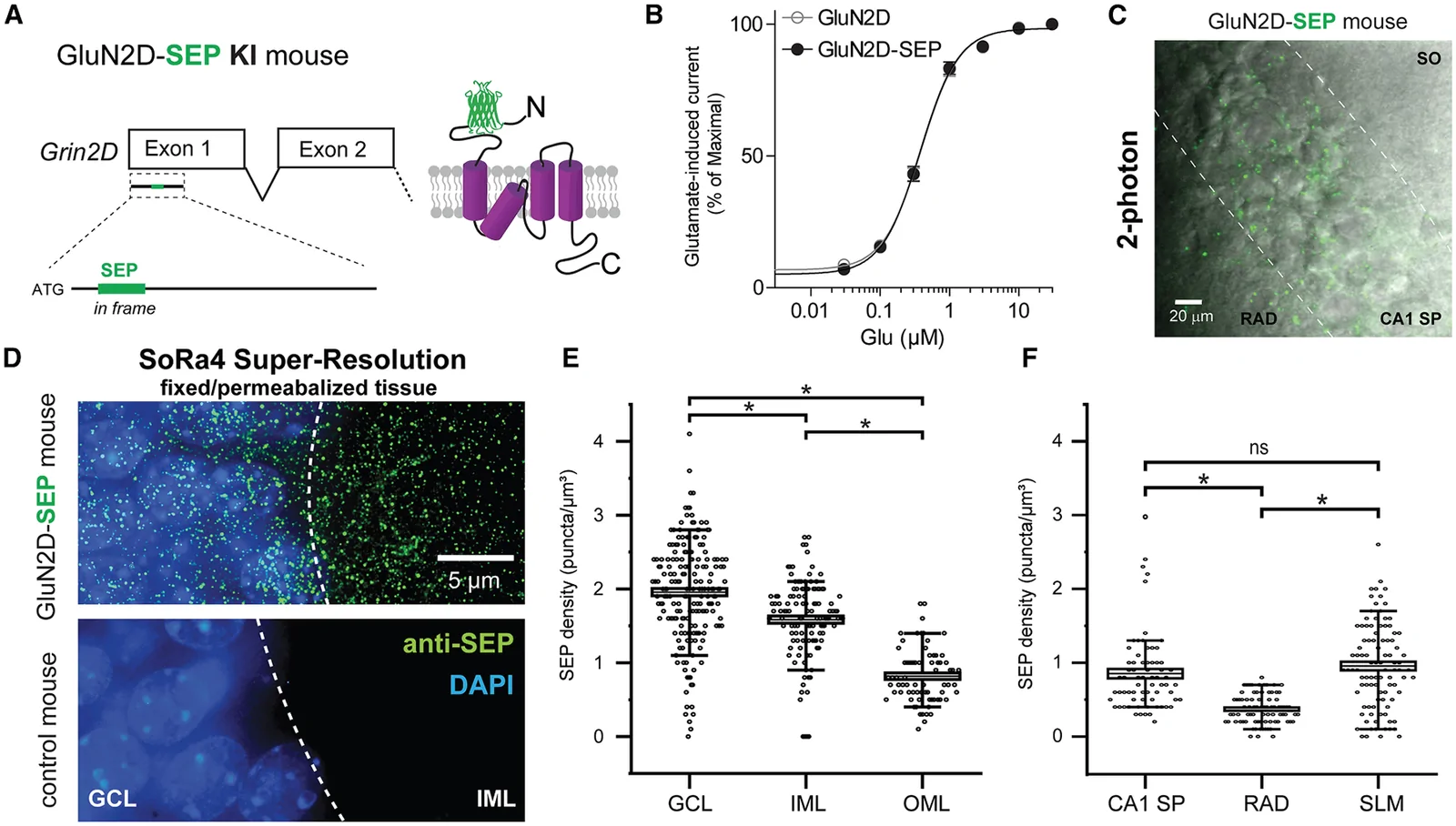
Characterization of GluN2D-SEP signal in hippocampus (A) The design for the knock-in of SEP (GluN2D-SEP mice) into coding exon-1 of the endogenous *Grin2d* gene is illustrated. SEP was inserted in-frame after the signal peptide in the extracellular N terminus (Figure S1). (B) Superimposed concentration-response curves for glutamate activation of WT GluN1/GluN2D and GluN1/GluN2D-SEP show that in-frame insertion of SEP does not change glutamate EC_50_. See Figure S2 for further characterization of GluN1/GluN2D-SEP. Data points are shown as mean ± SEM. (C) Native 2-photon green fluorescence signal in the CA1 of a living acute slice from GluN2D-SEP mouse hippocampus. Green puncta resembling synapses surround neuronal cell bodies. RAD-*stratum radiatum*, CA1 SP-CA1 *stratum pyramidale*, *stratum oriens* (SO). (D) *Left to right* Super-resolution images from the dentate GCL-IML border of GluN2D-SEP and WT mice stained with an anti-GFP antibody and DAPI (scale bars, 5 μm). SEP puncta density was quantified in layers of the dentate gyrus and the CA1. Granule cell layer (GCL), inner molecular layer (IML). (E and F) Quantification of puncta is shown by region; outer molecular layer (OML), stratum lacunosum-moleculare (SLM) (dentate:one-way ANOVA F = 36.7,*p* < 0.0001, Tukey Post hoc, *p < 0.0001,*n* = 3 animals; CA1 One-way ANOVA F = 97.3,*p* < 0.0001, Tukey Post hoc, *p < 0.0001,*n* = 3 animals). All data points are shown as well as mean ± STD.

To explore the endogenous expression pattern of GluN2D, we developed a knock-in mouse with super-ecliptic pHluorin (SEP) fused in-frame to the extracellular GluN2D N-terminal (Figure 1A; Figure S1), allowing for the detection of GluN2D surface expression.^11^ Inclusion of SEP did not alter NMDAR glutamate (Figure 1B) or glycine potency, Mg^2+^ block, desensitization, or deactivation time course (Figure S2). We examined the spatial distribution of endogenous GluN2D-SEP *in vivo*, focusing on the dentate gyrus (DG) and CA1 cell body layers in the hippocampus. In 2-photon imaging experiments, we observed a lack of labeling in somatic or dendrite-like structures. Rather, puncta surrounding the somas of excitatory neurons (Figure 1C) were present in both DG and CA1. We subsequently performed super-resolution (~120 nm xy-resolution) imaging of hippocampal slices from PFA-perfused mice. Green puncta were observed in GluN2D-SEP sections stained with anti-GFP to label SEP, which were absent in sections from WT controls imaged using identical settings (Figure 1D; Figure S3). SEP puncta were detected using automated analysis (Figure S3) across the DG, CA1 *str.pyramidale*, and adjacent regions. In DG, GluN2D-SEP density was highest in the dentate granule cell layer (GCL), followed by reduced densities in the inner and outer molecular layers. In CA1, GluN2D-SEP density was highest in *str*.*pyramidale* and *str.lacanosum moleculare*, with respect to *str.radiatum* (Figures 1E and 1F). SEP puncta identified by immunostaining with anti-GFP and anti-PV antibodies were co-localized (Figure S3).

**Figure 2.**
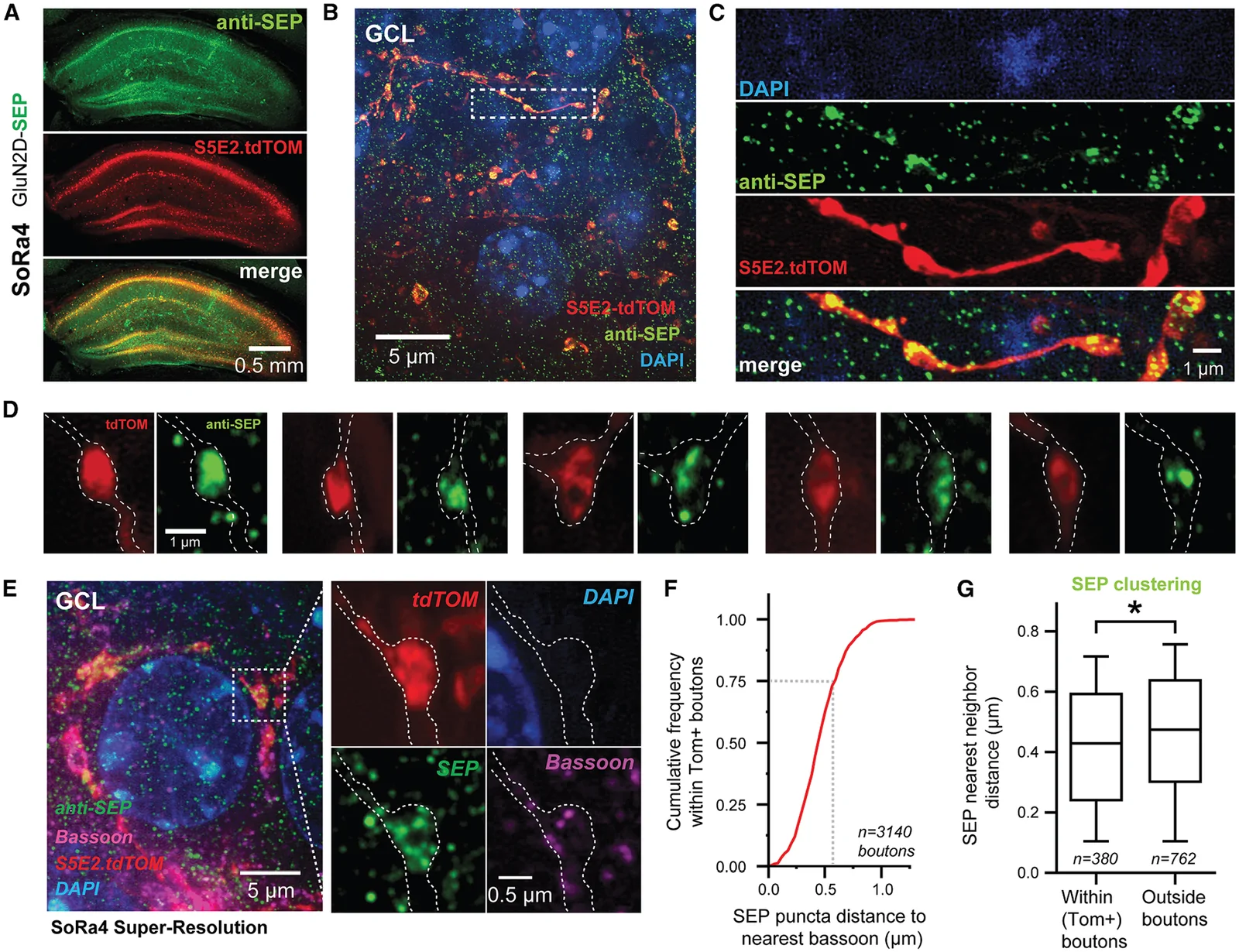
Enrichment of GluN2D-SEP signal in PV-interneuron basket boutons (A) SEP-GluN2D mice were stereotactically injected into the hippocampus with the PV-interneuron-selective enhancer AAV.S5E2.tdTom to examine potential overlap of perisomatic GluN2D-SEP with PV-interneuron basket axons. Widefield hippocampal images from an S5E2.The tdTom-injected SEP-GluN2D mouse is shown. (B–F) Multiple images of sections from the dentate GCL (B), with a zoomed example of a PV-axon (C) from PFA-perfused S5E2.tdTom-injected mice stained for anti-GFP to evaluate co-expression. The S5E2.tdTom signal corresponds with PV-basket axons and boutons, which surround GC somas (DAPI). Conglomeration of high-density GluN2D-SEP puncta with PV-axon swellings is notable. (D) High-resolution images illustrate co-localization of GluN2D-SEP within PV-containing boutons. (E) Mice were injected and sections treated as in (C), but also co-stained with an anti-Bassoon antibody to examine the relationship between PV-bouton swellings, presynaptic active zones, and SEP using SoRa4 spinning disk microscopy. (F) SEP puncta show preferential clustering within tdTOM+ processes, defined by a significantly shorter nearest neighbor (NN) distance between SEP puncta located in the close vicinity (<0.5 μm) of tdTOM+ bassoon puncta, in contrast to SEP puncta located outside these processes. Bassoon and SEP puncta nearest neighbor distance was quantified on three granule cell layer z-stacks from three GluN2D-SEP mice. (G) Quantification of automatically identified SEP puncta within and outside of tdTom+ PV-boutons (0.43 ± 0.01 vs. 0.47 ± 0.01, *n* = 380 and 762, for tdTOM+ SEP puncta vs. tdTOM- SEP puncta, respectively, two sample *t* test: *p* = 0.0015, t(df) = −3.17(1140)). Box plots show the mean, 25th-75th quartile, and STD (error bars).

As *Grin2d* mRNA is highest in PV-interneurons in hippocampus,^12^ we focused on the dentate GCL and CA1 s*tr.pyramidale* layers, given the presence of PV-interneuron somas and their perisomatic-targeting axons in these regions.^4^ To examine whether SEP puncta were associated with PV-interneuron synapses, we injected an adeno-associated virus (AAV) harboring the PV-selective S5E2 enhancer^13–17^ into GluN2D-SEP mice to drive red fluorophore expression (Figure 2A; Figure S4). We utilized immunohistochemistry and SoRa4 super-resolution imaging to quantitatively evaluate the relationship between SEP, PV (S5E2-labeled) axons, and bassoon. In S5E2.tdTom-injected sections, red bouton swellings and connecting axons surrounded DAPI-labeled granule cells (GCs) in the dentate, as expected for PV-basket synapses (Figures 2B and 2C). Although resolution-limit-sized SEP puncta were often found outside of PV-axons, PV-bouton swellings were clearly associated with denser SEP signals (Figures 2C–2E and 2G). To quantify whether GluN2D-SEP is localized near PV-presynaptic release machinery, we measured the distance of each SEP puncta from the nearest bassoon puncta in 1,000s of tdTom-labeled PV-boutons (Figures 2E and 2F) in the dentate GCL. SEP puncta were universally less than 1.0 μm away from bassoon puncta, with most falling <500 nm away (Figure 2F). Despite incomplete labeling of all PV-axons, we still resolved a significantly higher density of SEP puncta clustering within red PV-boutons than in unlabeled areas (Figure 2G). We also observed similarly strong co-localization of SEP using an anti-parvalbumin antibody (Figure S3E). Altogether, these data indicate clustered enrichment of GluN2D-NMDARs at PV-basket synapses.

### Subcellular molecular targeting via the GluN2D C terminus

**Figure 3.**
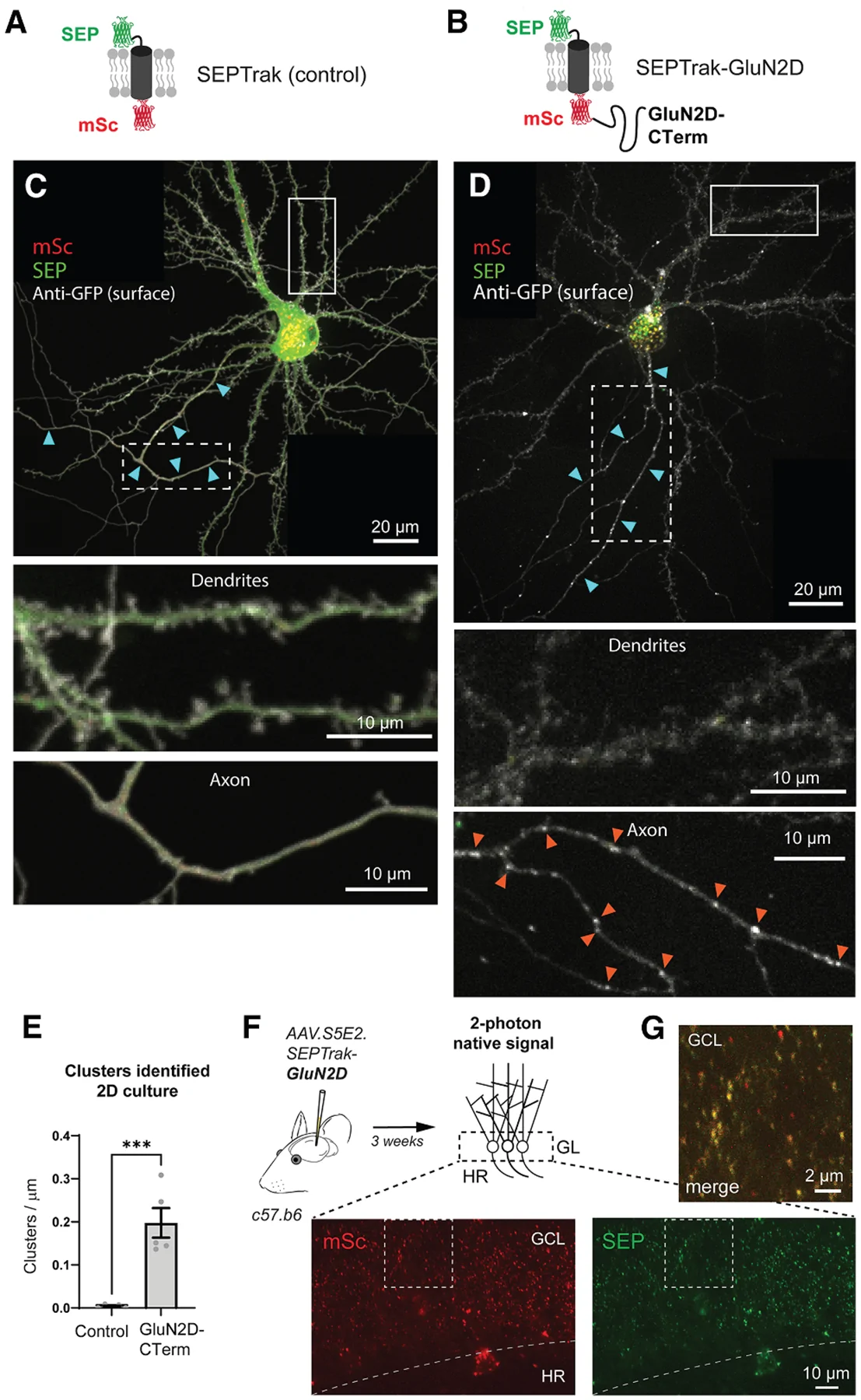
The GluN2D C-terminal is sufficient for axonal targeting (A and B) SEPTrak and SEPTrak-GluN2D were generated by placing the GluN2D C-terminal on the intracellular side of a single-pass transmembrane domain derived from the PDGF receptor. mScarlet and SEP were fused in-frame in the intra- and extracellular sequences, respectively, to visualize surface targeting. Expression *in vitro* was accomplished by transient transfection, and *in vivo* was driven by an AAV-delivered S5E2 enhancer for PV-interneuron expression. (C) Cultured hippocampal neuron transfected with SEPTrak control lacking the GluN2D C-terminal. Note the surface SEP signal (green) was also visualized by labeling with a dye-conjugated anti-GFP nanobody (gray). A section of the axon is indicated by the white dashed line and expanded below. Both SEP and anti-GFP signals are evenly distributed throughout all compartments of the neuron. Individual confocal image fields were stitched together to cover a large field of view at high resolution, displaying both somatodendritic and axonal compartments. Black regions represent incomplete imaging coverage in the final stitched images. (D) Cultured hippocampal neuron transfected with SEPTrak-GluN2D with the GluN2D C-terminal domain fused in frame. Note the prominent surface puncta localized throughout the axonal and presynaptic terminal compartments. The image is a stitched composite of several individual fields of view. (E) Quantification of axonal puncta across multiple experiments for SEPTrak control and SEPTrak-GluN2D (Two Sample t-test, ***p < 0.0001, n=3 animals). All data points are shown as well as mean ± STD. (F) Representative 2-photon images of SEPTrak-GluN2D expression in acute hippocampal slices following transduction with AAV directed to PV-interneurons by the S5E2 enhancer. Intracellular mScarlet and extracellular SEP are shown in different channels. GCL-granule cell layer, HR-hilar region. (G) Superimposed and zoomed image from within the GCL region in the boxed region within F. The result was representative across multiple injections (*n* = 3 mice; Figure S5).

When compared to other GluN2 subunits, the GluN2D sequence is unique, especially with respect to its C-terminal, which likely encodes trafficking information.^18–20^ Thus, we tested whether the GluN2D C-terminal alone is sufficient to specify trafficking of a generic membrane-associated protein. We built a modified pDisplay plasmid expressing the GluN2D C-terminal on the intracellular side of a single-pass transmembrane domain (referred to as SEPTrak-GluN2D). mScarlet and SEP were fused in-frame in the intra- and extracellular sequences, respectively (Figure 3A) to visualize surface (SEP) and total (mScarlet) expression patterns in neurons. A control version lacked the GluN2D C-terminal (Figure 3A). Cultured rat hippocampal neurons transfected with our control SEPTrak vector show uniform surface expression throughout all neuronal compartments, without noticeable accumulation or clustering in any subcellular compartment (Figures 3B and 3C). This is consistent with the well-known properties of fluorescent proteins encoded by SEPTrak. Conversely, inclusion of the intracellular GluN2D C-terminal on SEPTrak produced accumulation of strong punctate fluorescence in neurites (Figure 3D). Prominent puncta were apparent in both the axonal and presynaptic compartments (Figure 3D; Figure S5). There was a striking difference in the number of axonal puncta detectable between control SEPTrak and that containing the GluN2D C-terminal fused in frame (Figure 3E).

Our *in vitro* characterization of SEPTrak-GluN2D showed that the GluN2D C-terminal directs proteins to punctate axonal domains. To assess the ability of the GluN2D C-terminal to control subcellular localization in interneurons *in vivo*, we placed the SEPTrak-GluN2D construct under control of the PV-selective S5E2 enhancer within an AAV vector, and injected AAVs harboring this cassette into the hippocampus of WT mice (Figure 3F). Strong somatodendritic signatures were rarely seen, but when encountered, displayed a much higher mScarlet signature compared to surface (SEP) signal (Figure S5), suggesting diminished somatodendritic surface expression. By contrast, punctate SEPTrak-GluN2D signals were readily detected throughout the GCL (Figures 3F and 3G), suggesting substantial surface enrichment in PV-interneuron axons. Together, these data suggest that GluN2D has sufficient targeting information within its intracellular C terminus to direct axonal expression in neurons *in vitro* and PV-interneurons *in vivo*.

We next explored the potential for the localization of full-length GluN2D at high spatial resolution using cultured rat hippocampal neurons transduced with extracellularly ALFA-tagged GluN2D (full-length cDNA) and live/surface labeling, using a Cy3-conjugated anti-ALFA nanobody. We did not observe the colocalization of ALFA-GluN2D with the postsynaptic marker GluA1, suggesting that the post-synapse is not the primary destination for surface GluN2D-NMDARs in cultured neurons (Figure S6).

### GluN2D-NMDARs at PV-interneuron synapses dynamically modulate stochastic GABA release

Why might NMDARs be positioned at fast-spiking GABAergic basket synapses? One explanation is that the higher glutamate affinity of GluN2D, lack of desensitization, and slow kinetics are all suited to sense slower changes in ambient glutamate.^21–23^ Thus, the activation state of GluN2D would shift in response to changes in ambient glutamate, modulating stochastic and AP-evoked vesicular release of GABA via presynaptic depolarization, Ca^2+^ entry, and/or metabotropic signaling,^24–26^ thereby coupling inhibitory dynamics to excitatory network activity. This local axonal control, referred to as analog signaling,^27,28^ could reflect a paracrine mechanism mediated by glutamate released from other cells.^29^ However, evidence for robust mechanisms that engage axonal analog signaling in mature interneurons^30^ is lacking.

**Figure 4.**
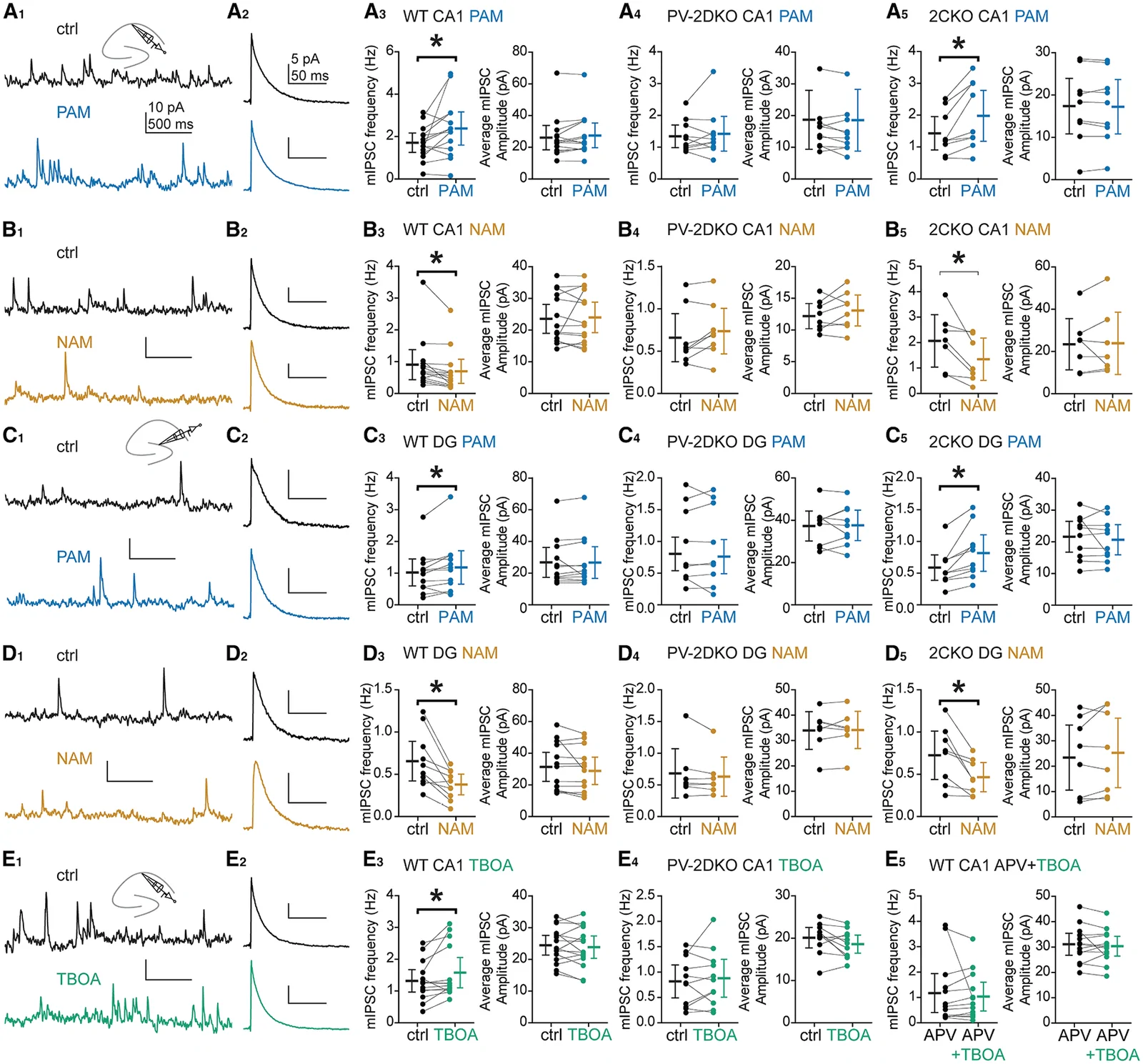
GluN2D-NMDARs in PV-interneurons bidirectionally control the rate of stochastic GABA release (A) Effect of GluN2D-selective positive allosteric modulator (+)EU1180–453 (PAM) on stochastic, non-action potential-evoked GABA release (mIPSCs) in adult hippocampal slices. (A1) Current recording under voltage clamp (+10 mV) is shown for control (black) from a CA1 pyramidal cell before and after application of (+)EU1180–453 (blue). mIPSCs were outward due to CsCl internal solution and holding the membrane potential at the AMPA receptor reversal potential (Methods). (A2) Mean mIPSC waveform. (A3) The frequency and amplitude from individual cells are shown. (A4) Lack of effect of 10 μM (+)EU1180–453 on slices from PV-GluN2D knockout (PV-2DKO) but not GluN2C knockout mice (2CKO,A5). For frequency WT CA1 PAM *p* = 0.035, PV-2DKO *p* = 0.54, 2CKO *p* = 0.010; for amplitude WT *p* = 0.17, PV-2DKO *p* = 0.21, 2CKO *p* = 0.60. (B) Same as above for A, except 1 μM of (S)-DQP-997–74 (NAM) was used. For frequency WT CA1 NAM *p* = 0.018, PV-2DKO *p* = 0.10, 2CKO *p* = 0.029; for amplitude WT *p* = 0.72, PV-2DKO *p* = 0.24, 2C KO *p* = 0.85. (C and D) Same as A except recordings were from dentate (DG) granule neurons in response to a PAM (C) or a NAM (D). For frequency WT DG PAM *p* = 0.034, PV-2DKO *p* = 0.87, N2C KO *p* = 0.0053; for amplitude WT *p* = 0.94, PV-2DKO *p* = 0.84, 2CKO *p* = 0.47. For frequency WT DG NAM *p* = 0.0039, PV-2DKO *p* = 0.20, 2CKO *p* = 0.012; for amplitude WT *p* = 0.30, PV-2DKO *p* = 0.83, 2CKO *p* = 0.23. (E) Blocking glutamate uptake with 1 μM TFB-TBOA increased the mIPSC frequency in CA1 pyramidal cells, and this effect was absent in slices from PV-2DKO mice (E4) or when 200 μM DL-APV was present (E5). Data are mean (large hash) ± STD (small hash); * indicates *p* < 0.05 (paired *t* test). For frequency WT TBOA *p* = 0.040, WT TBOA+APV *p* = 0.56, and PV-2DKO *p* = 0.49; for amplitude WT TBOA *p* = 0.62, WT TBOA+APV *p* = 0.46, and for PV-2DKO TBOA *p* = 0.09 (Tables S1 and S2 for statistics).

With this framework in mind, we performed patch-clamp recordings from dentate GCs and CA1 pyramidal neurons in 8–12 week hippocampal slices using highly GluN2D-selective pharmacological probes to assess potential NMDAR control of GABA release. First, we recorded miniature inhibitory postsynaptic currents (mIPSCs) under voltage clamp in TTX (1 μM). We evaluated the effect of GluN2C/D-selective PAMs and negative allosteric modulators (NAMs) on mIPSC frequency and found that GluN2D modulation bidirectionally controls the rate of stochastic GABAergic vesicular release. The GluN2C/D-selective PAM (+)EU1180–453^9,31^ potentiates the response of GluN2C- or GluN2D-NMDARs several-fold with a low micromolar EC_50_, and has no effect on AMPA, kainate, GABA, glycine, and over 40 other receptors, channels, and transporters.^9,31^ In addition, the NAM (S)-DQP-997–74 inhibits GluN2C- and GluN2D-NMDARs with an IC_50_ value of 50 nM, and is without effect on AMPA, kainate, GABA, glycine, nicotinic, and P2X receptors.^32^ (+)EU1180–453 (10 μM) application rapidly increased mIPSC frequency in both pyramidal cells and GCs (Figures 4A–4C), whereas 1 μM of (S)-DQP-997–74 reduced mIPSC frequency in both cell types (Figures 4B–4D). There was no effect on mIPSC amplitude or time course by either treatment. We used the two most common internal solutions for interneuron recording to ensure reproducibility, which gave identical results in terms of mIPSC sensitivity to a GluN2D-selective modulator (Figures S7A–S7C).

These pharmacological effects appear to be mediated by GluN2D-NMDARs on PV-interneurons, as modulator-induced mIPSC frequency changes were absent in experiments from conditional PV-GluN2D knockout mice (Figures 4A–4D). Drug effects were also not due to actions at GluN2C, as modulator-induced changes in mIPSCs were intact in global GluN2C KO mice (Figures 4A–4D).

To ensure these effects reflect drug modulation of presynaptic GluN2D, we performed two additional experiments. First, we recorded in DMSO vehicle for the full duration of the experiment to ensure there was no drift in mIPSC frequency confounding our results. We did not detect any significant difference in the mIPSC frequency during baseline or DMSO recording periods (Figure S7D). Second, we included MK-801 in the postsynaptic recording pipette solution, which should inhibit NMDAR-mediated conductances in the postsynaptic CA1 pyramidal cells. Treatment of slices with 10 μM (+)EU1180–453 increased mIPSC frequency to the same extent in CA1 pyramidal cells filled with MK-801 as normal recording solution (Figure S7E), ruling out postsynaptic NMDAR involvement.

Our results showing effects of a GluN2D-selective inhibition on mIPSC frequency suggest that basal glutamate levels around the inhibitory terminal are sufficient to activate GluN2D. To test whether increasing endogenous glutamate levels could further engage GluN2D-dependent control of GABA release, we blocked glutamate uptake pharmacologically during recordings. We found that 1 μM TFB-TBOA increased mIPSC frequency, consistent with the idea that changes in extrasynaptic glutamate levels^33^ can modify vesicular GABA release (Figure 4E). This effect was absent in the presence of D,L-2-amino-5-phosphonovalerate (DL-APV) (200 μM), confirming dependence on NMDARs. Furthermore, there was no effect of TFB-TBOA on mIPSCs recorded from CA1 pyramidal cells in slices obtained from PV-GluN2D knockout mice, confirming that the increased extracellular glutamate concentration secondary to impaired glutamate uptake acted on GluN2D-NMDARs. Together with data showing no effect of GluN2D potentiation on PV-interneuron somatic properties (Figure S4), these results are consistent with increased extracellular glutamate exerting its actions on mIPSC frequency through presynaptic GluN2D-NMDARs (Figure 4E; Figure S7E). These data suggest an unexpected and important role for GluN2D control of presynaptic GABA release from PV-interneurons in the hippocampus and potentially elsewhere, which have been hypothesized but not explicitly shown to possess GluN2D-NMDARs.^34,35^ Localization of GluN2D-NMDARs at PV-presynaptic sites could, in principle, account for these phenomena.

### GluN2D-NMDARs set basal release properties of PV-axons

**Figure 5.**
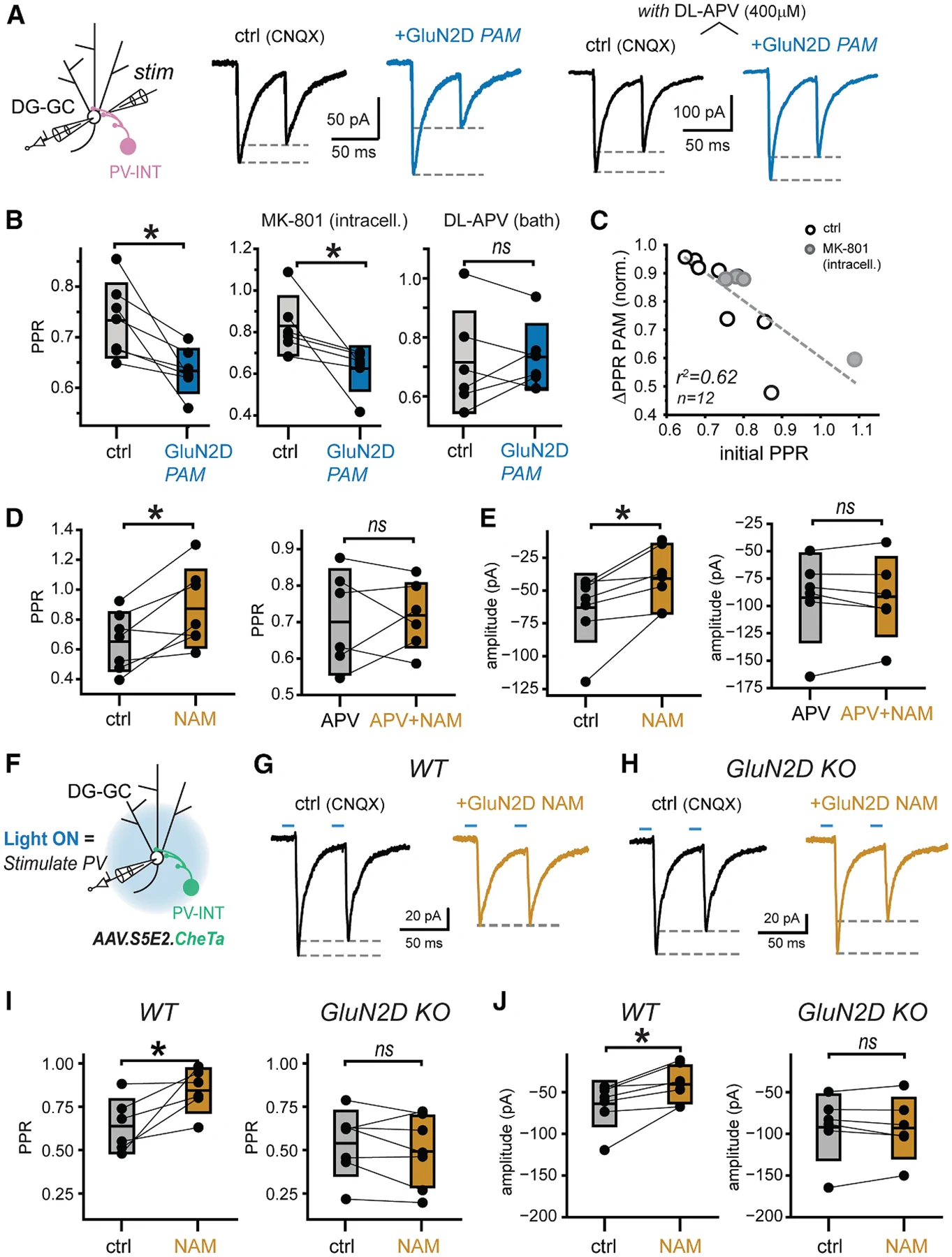
Effects of GluN2D-NMDARs on AP-evoked release at PV-synapses (A) The effect of 10 μM (+)EU1180–453 (GluN2D PAM) on paired AP-evoked inhibitory release is shown, with a cartoon illustrating the experimental approach (*left*). Representative evoked IPSCs are shown (*right*) from whole-cell dentate GC current recordings coupled with local stimulation (TTX omitted). Inclusion of 400 μM APV eliminated the effect of the GluN2C/GluN2D-selective PAM (+)EU1180–453. (B) Within-cell paired data summary of the PAM effect on paired-pulse ratio (PPR) as described in (A), for experiments in which MK-801 (50 μM) was included in the patch pipette, and for experiments where DL-APV (400 μM) was bath applied during the experiment. Boxplots include the average (*middle line*) and the SD. For PPR WT DG PAM *p* = 0.017, MK-801 *p* = 0.046, DL-APV *p* = 0.67. (C) Summary data depict the relationship between initial absolute PPR (lower values putatively correspond to PV-synapses with higher initial basal release probability) and the PPR change following GluN2D PAM application. The correlation indicates that GluN2D potentiation was stronger for individual PV synapses with lower basal release probability (Figure S4). (D and E) Within-cell paired data summary of 1 μM GluN2D NAM (S)-DQP-997–74 effect on PPR and the amplitude of the 1^st^ evoked IPSC. Where noted, DL-APV (400 μM) was bath applied during the experiment. Boxplots include the average (*middle line*) and SD. *indicates *p* < 0.05 from a paired *t* test. For PPR WT DG NAM *p* = 0.013, APV+NAM *p* = 0.84, amplitude WT DG NAM *p* = 0.012, APV+NAM *p* = 0.85. (F) Schematic diagram illustrates optogenetic experiments in which an AAV drives the CheTa version of channelrhodopsin under the S5E2 enhancer. (G and H) Current responses recorded from dentate granule cells in response to paired blue light stimuli show evoked IPSCs arising from GABA release from PV-interneuron terminals. Recordings were made from slices prepared from WT and PV-GluN2D knockout mice. (I) Within-cell paired data summary of the GluN2D NAM (S)-DQP-997–74 effect on PPR in WT and PV-GluN2D knockout slices. Data are mean ± STD and * indicates *p* < 0.05 (paired *t* test). For PPR WT DG NAM *p* = 0.037, PV-GluN2D KO NAM *p* = 0.25. (J) Within-cell paired data summary of the GluN2D NAM (S)-DQP-997–74 effect on the amplitude of the 1^st^ evoked IPSC in WT and PV-GluN2D knockout slices. * indicates *p* < 0.05 from a paired *t* test. For amplitude WT DG NAM *p* = 0.032, APV+NAM *p* = 0.91.

To further evaluate the effect of GluN2D modulation at PV-synapses, we recorded from postsynaptic dentate GCs during extracellular stimulation immediately adjacent to the soma (Figure 5A) to evoke synchronous GABA release from PV-interneurons. Evoked IPSCs in these experiments originated from PV-basket synapses, as they were fully blocked by ω-agatoxin (Figure S4).^36^ As expected for a presynaptic effect, application of the GluN2D-selective PAM (+)EU1180–453 reduced the paired-pulse ratio without altering the IPSC time course (Figure 5B, 16 ± 1.2 vs. 16 ± 0.94 ms for control and PAM, respectively, *p* = 0.88, Student’s paired *t* test, *n* = 6). This effect was similar in experiments in which MK-801 (50 μM) was included in the pipette to block postsynaptic NMDAR conductance (Figure 5B). However, the amplitude of the first evoked IPSC was unchanged (Figure S4B) after (+)EU1180–453 treatment, perhaps a result of a ceiling effect or vesicular depletion at these high P_r_ release sites.^37,38^ Nonetheless, the ΔPPR magnitude after (+)EU1180–453 correlated with the initial basal paired pulse ratio (PPR) for each recording (Figure 5C), reinforcing the notion that GluN2D activity influences release at basket synapses in a release-probability-dependent manner. The (+)EU1180–453 effect on PPR requires occupancy of the GluN2D glutamate binding site, as it was abolished by the competitive antagonist D-APV (400 μМ; Figure 5D). The PPR change after (+)EU1180–453 was not due to passive depolarization originating from PV-somas, as it did not alter membrane potential or intrinsic properties (Figures S4C and S4D). We next performed the same experiment using the NAM (S)-DQP-997–74, which had a striking effect on the PPR at the DG GC synapse, transforming its reliably depressing nature into a nearly facilitating phenotype. This PPR change was also accompanied by a reduction in the amplitude of the first IPSC (Figures 5D and 5E) without a change in IPSC time course (9.0 ± 0.54 vs. 8.6 ± 0.84 ms for control and NAM, respectively, *p* = 0.34, Student’s paired *t* test, *n* = 7). No changes in PPR or amplitude were seen following (S)-DQP-997–74 application when D-APV was included in the bath (Figures 5D and 5E). This again indicates that glutamate binding to GluN2D receptors is necessary to observe NAM-induced synaptic effects.

To ensure the effects on AP-evoked GABA release of GluN2D-NMDARs modulation in PV-interneuron terminals were not confounded by off-target effects of extracellular stimulation, we used a PV-interneuron optogenetic approach to selectively stimulate PV-axons alone. An AAV expressing S5E2.CheTa, which allows single AP fidelity in PV-interneurons with short blue light pulses,^17^ was injected into the dentate of either WT or PV-GluN2D knockout mice (Figure 5F). Three weeks thereafter, we recorded from postsynaptic dentate GCs and performed paired-pulse optical stimulation to evoke AP firing in presynaptic PV-interneurons (Figure 5G). In WT mice, (S)-DQP-997–74 application increased the PPR, alongside a reduction in the amplitude of the 1^st^ IPSC (Figures 5I and 5J). Both the PPR and amplitude effects following NAM (S)-DQP-997–74 were absent in mice lacking GluN2D in PV-interneurons (Figures 5I and 5J). Together, these results indicate a presynaptic action of GluN2D in regulating AP-evoked inhibitory neurotransmission.

To examine whether GluN2D modulation affects subthreshold Ca^2+^ levels in PV-basket boutons, we performed population presynaptic PV-Ca^2+^ imaging (1 μM TTX, 2 mM Ca^2+^, 1 mM Mg^2+^) with GCaMP6f, before and after local application of (+)EU1180–453 (Figures S4E and S4F). Despite evidence that GluN2D potentiation enhances stochastic GABA release, we could not resolve changes in subthreshold Ca^2+^ at PV-boutons, reminiscent of high-resolution Ca^2+^-imaging experiments in cerebellar interneuron axons.^39^ It remains possible that steady-state Ca^2+^ changes were below the resolution of our approach, especially if Ca^2+^ entry via GluN2D-NMDARs occurs during rare, brief channel openings.^21,23,40^ However, the lack of detectable changes in terminal basal Ca^2+^ concentration leaves open the possibility that GluN2D-NMDARs may signal metabotropically at PV synapses.^41^

### The role of GluN2D in the therapeutically relevant effects of ketamine

Deficits in PV-interneurons have been reported in postmortem studies of patients with schizophrenia,^42^ and alterations in gamma oscillations arising in part from PV-interneuron activity^9,10^ are an electrophysiological signature of this condition.^43^ In addition, selective inhibition of PV-interneurons in the frontal cortex can bring about antidepressant effects in animal models of depression,^44^ and PV-interneuron regulation contributes to the antidepressant effects of ketamine via NMDARs.^45–47^ In the context of treatment-resistant depression, ketamine (effective by yet unclear mechanisms)^2^ is hypothesized to diminish GABA release from PV-basket cells, which triggers downstream changes in excitatory synaptic transmission that may underlie improvement in clinical symptoms.^48–50^ However, the idea that GluN2D-NMDARs at basket cell GABAergic terminals are involved in ketamine’s actions has not been previously proposed. To evaluate the potential role of presynaptic NMDARs in ketamine’s actions, we recorded mIPSCs in the presence of low-concentration ketamine, similar to predicted plasma levels (~0.5 μM) for ketamine doses (0.2–0.5 mg/kg^51,52^) used in treatment-resistant patients.

**Figure 6.**
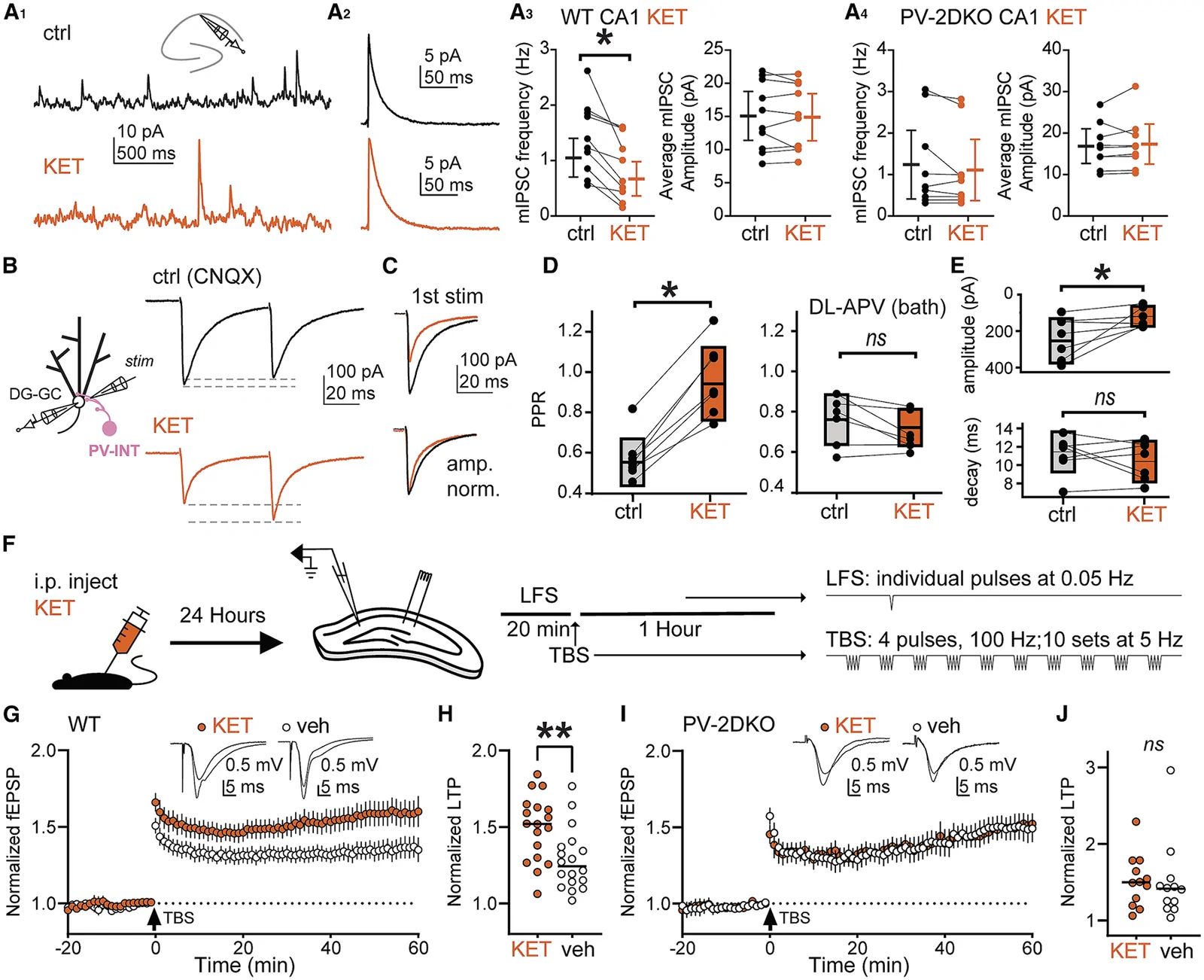
Effects of low-concentration ketamine on presynaptic PV-synaptic release properties and plasticity (A) Effect of 1 μM ketamine on mIPSCs in adult hippocampal slices. (A1) Voltage clamp current recording from a CA1 pyramidal cell before (black) and after application of ketamine (vermillion). mIPSCs were outward due to the internal solution and holding at the AMPA receptor reversal potential. (A2) Mean mIPSC waveform. (A3) Summary for the ketamine effect on mIPSC frequency and amplitude. (A4) mIPSCs recorded from slices made from a PV-GluN2D knockout (PV-2DKO) mouse were insensitive to ketamine. Data are mean ± STD and * indicates *p* < 0.05 (paired *t* test). For frequency WT *p* = 0.0024, PV-2DKO *p* = 0.17, and for amplitude WT *p* = 0.62, PV-2DKO *p* = 0.47 (Tables S1 and S2 for statistics). (B) Effect of 1 μM ketamine on paired AP-evoked inhibitory release. Cartoon depiction of experimental approach in dentate GC layer (*left*) and representative evoked IPSC recordings (*right*) from dentate GCs held under voltage clamp and subject to local stimulation of PV-interneuron axons. (C) Response waveforms are superimposed for the first evoked IPSC in control and after ketamine application (*top*). The same IPSCs are shown with amplitudes (*bottom*). (D) Within-cell paired data summary of the ketamine effect on PPR (paired-pulse ratio) (*left*), including experiments where MK-801 was included in the postsynaptic recording pipette solution or DL-APV (400 μM) was included in the background (*right*) during the experiment. Data are mean ± STD and * indicates *p* < 0.05 (paired *t* test). For PPR ketamine *p* < 0.0001, DL-APV ketamine PPR *p* = 0.0529. (E) Within-cell paired data summary of the ketamine effect on IPSC amplitude and time course measured as the tau for a single exponential for the 1^st^ response to stimulation. Data are mean ± STD and * indicates *p* < 0.05 (paired *t* test). For amplitude *p* = 0.046, decay *p* = 0.41. (F) Experimental design for the evaluation of durable effects of *in vivo* ketamine (KET) measured 24 h post-injection on LTP induced by a theta-burst protocol. 4–12-week-old mice were i.p.-injected with sterile saline or 20 mg/kg racemic ketamine, and 24 h post-injection, hippocampal slices were isolated, and LTP was induced in the CA1 region using a theta-burst stimulation protocol applied to the Schaffer collaterals. (G–J) Ketamine (20 mg/kg; 24 h post-i.p. injection) enhanced the magnitude of LTP induced in WT mice compared to vehicle (G and H vehicle 1.28 ± 0.05 *n* = 18, ketamine 1.49 ± 0.05 *n* = 18, *p* = 0.005), but did not alter LTP level in mice lacking GluN2D in PV-interneurons (I and J, vehicle 1.50 ± 0.14 *n* = 12, ketamine 1.52 ± 0.09 *n* = 12, *p* = 0.971). *Inset*, representative recordings from vehicle- or ketamine-treated mice before and after LTP induction. Normalized EPSP plots show group mean ± SEM, summary normalized LTP plots include individual slice responses and the group average (*middle line*).

Figure 6A shows that 1 μM ketamine applied to hippocampal slices acutely reduced the mIPSC frequency, without effect on amplitude, in CA1 pyramidal cells. Strikingly, this effect was absent in slices from mice lacking GluN2D in PV-interneurons (Figure 6A). To further confirm that ketamine acts presynaptically, we performed extracellular stimulation while recording from postsynaptic GCs to evoke PV-mediated IPSCs (Figures 6B and 6C). Ketamine (1 μM) had a robust effect, transforming the reliably depressing PV-GC synapse to a near-facilitating phenotype (Figure 6D). This ketamine-induced alteration in short-term plasticity was NMDAR-dependent, as it was abolished by DL-APV (400 μМ) (Figure 6D). Consistent with a presynaptic effect, ketamine did not affect the time course of the IPSC (Figure 6E). Together, these data provide evidence that GluN2D in PV-interneurons is a target for ketamine, and demonstrate that blocking their activity alters the basal synaptic properties (high release probability; depressing short-term plasticity) of these cells.

An important aspect of ketamine’s clinically useful actions is hypothesized to involve durable changes in long-term synaptic plasticity and altered synaptic signaling that persist long after ketamine is eliminated from the body. Studies in mice indicate that 24 h after ketamine administration, theta-burst-induced long-term potentiation (LTP) in CA1 pyramidal cells in acutely prepared slices is enhanced compared to vehicle.^53,54^ We confirmed this by administering ketamine (20 mg/kg i.p.) or vehicle 24 h before the preparation of slices from WT mice and LTP induction (Figures 6F and 6G). This well-established, durable effect of ketamine on LTP was absent in mice lacking GluN2D in PV-interneurons (Figure 6H), indicating the necessity of GluN2D-NMDARs expressed in PV-interneurons for the process.

### Transient inhibition of GluN2D produces a durable improvement of stress-related amotivation

**Figure 7.**
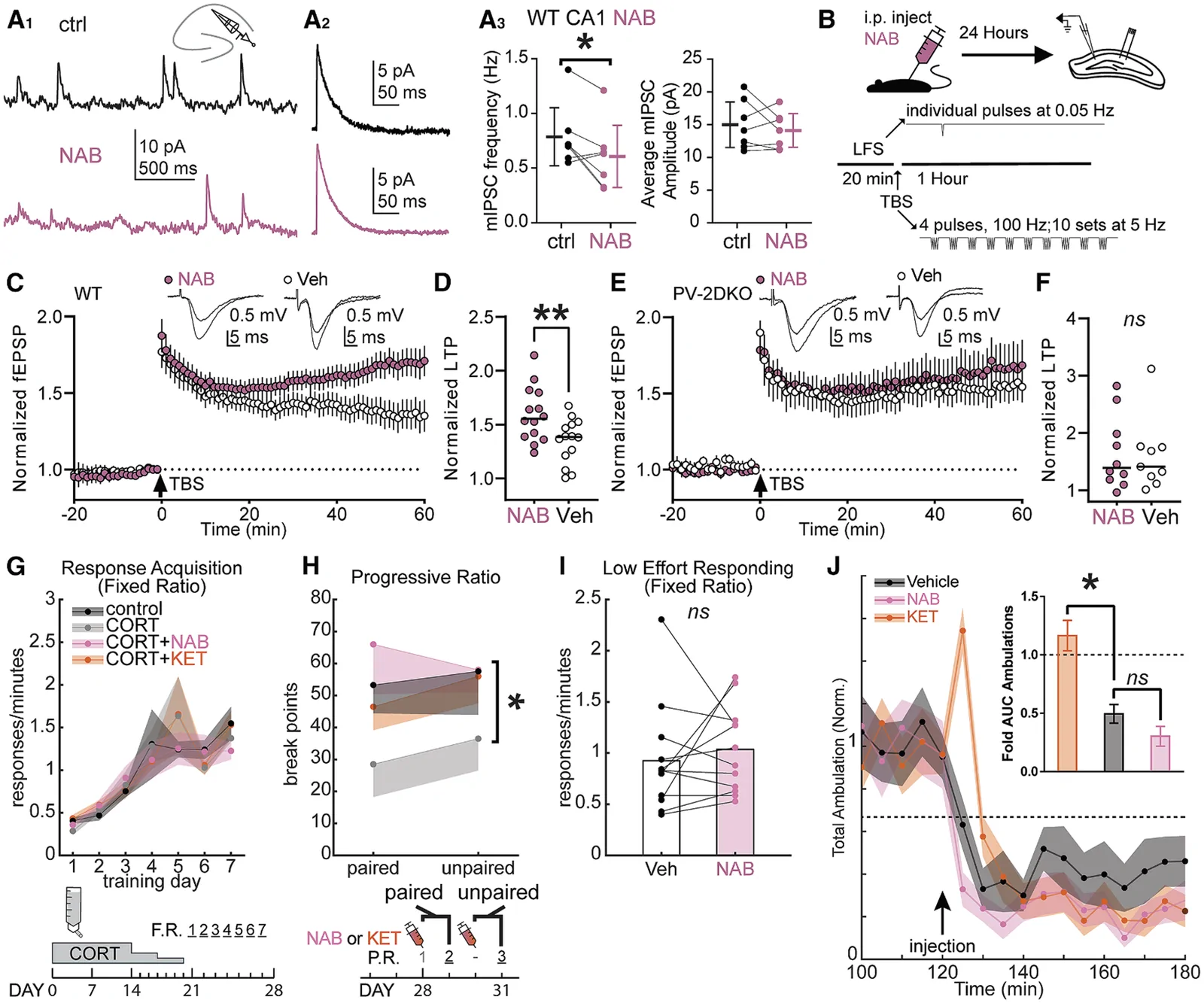
The GluN2C/GluN2D-selective inhibitor NAB14 has antidepressant-like properties indistinguishable from ketamine (A) Effect of 10 μM NAB14 on mIPSCs in hippocampal slices from adult mice. (A1) Voltage clamp current recording from a CA1 pyramidal cell before (black) and after application of 10 μM NAB14 (purple). mIPSCs were outward due to the internal solution and recording at the AMPA receptor reversal potential. (A2) Mean mIPSC waveform. (A3) Data summary for the NAB14 effect on mIPSC frequency and amplitude. Data are mean ± STD and * indicates *p* < 0.05 (paired *t* test). For frequency *p* = 0.047 and for amplitude *p* = 0.46, see Tables S1 and S2 for statistical parameters. (B) Experimental design for evaluation of durable effects of *in vivo* NAB14 measured 24 h post-injection on synaptic plasticity in hippocampal slices (LTP) induced by a theta-burst stimulation protocol. 4–12-week-old mice were injected i.p. with vehicle or 10 mg/kg NAB14, and 24 h post-injection, hippocampal slices were isolated and LTP induced in the CA1 region using a theta-burst stimulation protocol applied to the Schaffer collaterals. (C–F) NAB14 (10 mg/kg) enhanced the magnitude of LTP induced in WT mice with respect to vehicle, assessed 24 h post i.p.-injection (vehicle 1.6 ± 0.07 *n* = 14, NAB14 1.4 ± 0.05 *n* = 14, *p* = 0.0091). The *inset* shows representative recordings from vehicle- or NAB14-treated mice before and after LTP induction. (D) Summary plot of data from all slices; plots include average (*middle line*). (E) NAB14 (10 mg/kg) had no detectable effect on the magnitude of LTP induced in PV-GluN2D knockout mice with respect to vehicle, assessed 24 h post-i.p. injection (vehicle 1.7 ± 0.20 *n* = 10, NAB14 1.6 ± 0.21 *n* = 9, *p* = 0.882). The *inset* shows representative recordings from vehicle- or NAB14-treated mice before and after LTP induction; (F) Normalized EPSP plots show group mean ± SEM, summary normalized LTP plots include individual slice responses and the group average (*middle line*). (G) Mice were exposed to CORT in the drinking water and subsequently trained to respond for food rewards in the absence of CORT. Groups did not differ during initial training under low-effort conditions. Shaded lines represent group means ± SEMs. (H) Mice were tested on a progressive ratio schedule of reinforcement, in which the response demands progressively increased with each food reward delivery. Vehicle, ketamine, or NAB14 were administered i.p. after training as indicated in the timeline below. Mice were tested 24 h following drug/vehicle injections, aligning with the durable physiological consequences of these drugs. Mice were tested in two conditions, with the drugs paired with reward (“paired”) or when they were simply injected and left undisturbed for 24 h (“unpaired”). CORT decreased break point ratios, a depression-like response reflecting amotivation. NAB14 (10 mg/kg) recovered motivated responding and was indistinguishable from ketamine, regardless of whether the drugs were paired or unpaired with the food reward. Main effect of group F_(3,27)_ = 3.055, *p* = 0.045, no main effect of pairing condition and no interaction Fs < 1. Posthoc CORT vs. ctrl *p* = 0.039, CORT vs. CORT+NAB14 *p* = 0.008, CORT+NAB14 vs. CORT+ketamine *p* = 0.302. Ctrl *n* = 7, CORT *n* = 8, CORT+NAB14 *n* = 8, CORT+ketamine *n* = 8. Shaded lines represent group means (+SEMs). (I) NAB14 had no impact on low-effort responding (one response results in one reinforcer). Paired t_11_ = 0.675, *p* = 0.513, *n* = 12 tested in both conditions. Bars represent group means, and lines represent individual mice. (J) Homozygous PV-Cre mice were placed in an activity monitoring box, and locomotion was recorded in 5 min periods. 10 mg/kg NAB14, 10 mg/kg ketamine (in the same vehicle as NAB14), or vehicle were administered i.p. at the indicated time (red arrow). Data are the number of ambulations/5 min normalized to the pre-injection baseline; the mean number of ambulations is in Figure S8. Ketamine increased locomotion, as expected, while NAB14 mice did not differ from vehicle-treated mice. Data are mean ± SEM; *n* = 9 (vehicle), *n* = 18 (NAB = NAB14), *n* = 12 (KET = ketamine). ANOVA with Tukey’s multiple comparisons test; F_(2, 35)_ = 20.10 *p* < 0.0001, Dunnett’s multiple comparisons post hoc *p* = 0.0004 ketamine vs. vehicle, and *p* = 0.34 for NAB14 vs. vehicle.

To further assess whether the inhibition of GluN2D can replicate effects of ketamine, we next turned to the brain-penetrant GluN2C/D-selective negative allosteric modulator, NAB14. This well-characterized^55^ inhibitor is selective for GluN2C/D-NMDARs, with no effect on AMPA, kainate, GABA, glycine, nicotinic, serotonergic, or purinergic receptors.^55^ We first tested whether 10 μM NAB14 had the same effect on mIPSC frequency as (S)-DQP-997–74. NAB14 significantly reduced mIPSC frequency without any effect on amplitude (Figure 7A). We subsequently evaluated whether NAB14 inhibition of GluN2D produces a durable change in LTP assessed 24 h after *in vivo* administration. We repeated the previous LTP experiment using 10 mg/kg i.p. administration of NAB14 instead of ketamine. NAB14 at this dose is brain-penetrant (Table S3) and, like ketamine, has a brief half-life (30 min Table S3).^55^ Similar to the result with ketamine, we found that 10 mg/kg NAB14 (but not vehicle) delivered *in vivo* 24 h prior to the isolation of brain slices enhanced theta-burst-induced LTP in an identical fashion as ketamine (Figures 7B, 7C, and 7D). The effect of NAB14 on LTP was abolished in mice lacking GluN2D in PV-interneurons (Figures 7E and 7F).

Depression is a leading cause of disability worldwide because it causes profound amotivation, leading individuals to struggle to maintain employment and perform even day-to-day activities.^56^ Ketamine is a clinically useful antidepressant that, unlike traditional antidepressants, can rapidly alleviate depression symptoms. Our findings suggest that NAB14 should share this antidepressive property, perhaps with reduced undesirable side effects. To test this possibility, mice were chronically administered the primary stress hormone corticosterone (CORT) in their drinking water, which induces cellular, circuit-level, and behavioral attributes of depression, including reward amotivation.^57–59^ Next, mice were trained to nose-poke for food reinforcers (Figure 7G). Under low-effort conditions (one poke results in one food pellet as reward), CORT-exposed mice did not differ from controls (Figure 7G). Next, mice were shifted to a progressive ratio schedule of reinforcement, in which each reward required a progressively increasing number of responses (x+4). The break point ratio refers to the highest number of responses that the mouse is willing to perform and serves as an indication of reward motivation.^60^ As previously shown,^58,61–65^ CORT-exposed mice generated low break points, reduced motivation, and thus depressive-like response. These and other CORT-induced depression-like behaviors are sensitive to reversal by chronic (but not acute) treatment with traditional antidepressants.^61,63,66^ We first confirmed that acute ketamine recovers motivated responding, conferring rapid antidepressant-like properties (Figure 7H). Next, we demonstrated that acutely administered NAB14 (10 mg/kg i.p.) had identical antidepressive consequences (Figure 7H). Importantly, mice were tested 24 h following injections, aligning with the durable physiological consequences of these drugs. Moreover, mice were tested in two conditions, with drug administration paired with reward (“paired”) or when the mice were simply injected and left undisturbed for 24 h (“unpaired”). NAB14 under both conditions restored motivation, with treated mice responding to the same level as control mice that did not receive CORT. That these compounds need not be paired with reward to be effective suggests that antidepressant properties are associated with drug-induced neurosequelae, and not nuanced drug × reward interactions. Given the largely unknown behavioral consequences of NAB14, we also confirmed that it had no effects on low-effort responding (one poke results in one food pellet; Figure 7I).

Finally, to assess whether NAB14 blockade of GluN2D could mimic hyperlocomotion of ketamine, we assessed locomotor activity following habituation of animals in activity monitoring boxes. Ketamine (10 mg/kg) administered in the same vehicle produced a robust increase in locomotor activity. By contrast, 10 mg/kg NAB14 did not increase locomotor activity and was indistinguishable from vehicle, which modestly dampened locomotor activity (Figure 7J). These data show that transient inhibition *in vivo* of GluN2D by NAB14 produced a durable increase in synaptic plasticity (LTP) and rectified stress-induced amotivation without the side effect of increased locomotor activity.

## DISCUSSION

The most salient conclusion from this study is that GluN2D-NMDARs act presynaptically on GABAergic terminals of hippocampal PV-interneurons to regulate the probability of vesicular release in response to extracellular glutamate. In addition, we show that the GluN2D C-terminal contains the information needed to target transmembrane proteins to the presynaptic terminal. Furthermore, our data suggest that multiple effects of low-dose ketamine depend on GluN2D-NMDARs on PV-interneurons, and selective block of GluN2D reproduces durable effects of ketamine on plasticity and motivation in a model of depression. These conclusions, particularly the robust effect of GluN2D inhibition in a model of depression with strong face validity, have implications for interneuron function, circuit activity, and therapeutic approaches to regulate synaptic transmission in neuropsychiatric illness. These results suggest that axonal GluN2D-NMDARs on PV-interneurons are a therapeutic target available to ameliorate circuit dysfunction in both neuropsychiatric and neurological disease. Transient inhibition of GluN2D at these sites appears to have durable effects on behavior that persist after the modulator is cleared from the brain.

Previous studies have raised the possibility of presynaptic NMDARs^67^ in cerebellar,^68–70^ dorsal motor vagal,^71^ and neocortical neurons.^72,73^ Dubois^34^ used global GluN2D-knockout mice to suggest that GluN2D is involved in presynaptic GABA release in stellate cells. Vicini recorded single-channel currents with conductance levels that were consistent with GluN2C- or GluN2D-NMDARs in outside-out patches excised from abnormally enlarged GABAergic terminals on cultured cerebellar neurons.^70^ Other studies have shown PV-specific effects of GluN2D during development,^74^ consistent with GluN2D expression in PV-interneurons.^2^ Moreover, GluN2A/2B subunits have been identified at postsynaptic densities, but not presynaptic sites formed by PV-interneurons, using freeze-fracture replica labeling.^75^ Our study evaluates the subcellular distribution and function of GluN2D on mature PV-interneuron synapses in adult gray matter (Figures 1 and 2) using highly selective modulators to show that GluN2D-NMDARs regulate vesicular release of GABA from PV-interneurons. Importantly, our data are supported by experiments in mature WT and conditional PV-GluN2D knockout mice (Figures 4 and 5).

We also provide data showing that the GluN2D C-terminal contains information that allows subcellular targeting *in vivo* to axonal and presynaptic terminal locations. This is in striking contrast to previous studies of isolated C-terminal domains. For example, Standley^76^ showed that the GluN2B C-terminal contains information that targets that subunit to the postsynaptic density, but not the presynapse. In addition, a wealth of data exists showing posttranslational mechanisms regulate trafficking,^77^ incorporation, and retention of GluN2A/B-NMDARs in postsynaptic compartments.^78^ The appreciation of a presynaptic targeting domain within GluN2D provides a mechanistic rationale supporting the anatomical and functional results presented here.

Our finding that GluN2D-NMDARs in PV-basket cells control GABA release holds several important implications. First, our data show that multiple effects of low ketamine concentrations *in vitro* are, in part, mediated by GluN2D-NMDARs on PV-interneurons. In addition, low concentrations of extracellular glutamate, derived from either spillover from excitatory terminals,^79^ inhibitory terminals that co-release glutamate and GABA (CCK interneurons^80^), or astrocytic glutamate release,^81,82^ may enhance inhibitory control of pyramidal cell excitability by increasing the vesicular release of GABA without altering PV-interneuron firing. This could serve as a graded auto-inhibitory feedback system that adjusts activity by sensing fluctuations in extrasynaptic glutamate. In this respect, the lack of desensitization and high potency of glutamate at GluN2D-NMDARs^6,22,23,83^ render these receptors sensitive real-time reporters of glutamate concentration—ideal characteristics for this hypothetical role. Moreover, the low open probability^23,40^ ensures that GluN2D-NMDARs will not overwhelm presynaptic terminals with Ca^2+^, cations, or osmotic gradients. Thus, the properties of GluN2D seem honed by selection for a presynaptic role, unlike postsynaptically localized GluN2A/B containing NMDARs.

The mechanisms underlying the remarkably durable response of patients suffering from treatment-resistant depression to ketamine have stimulated an enormous amount of research. Multiple mechanisms have been proposed to underlie this effect, including the hypothesis that ketamine produces disinhibition, leading to enhanced synaptic connectivity through multiple adaptive processes.^48,49,84^ One common element of these hypotheses is the central role of PV interneurons. Our work shifts the paradigm from the postsynaptic regulation of NMDARs in these cells to presynaptic NMDAR-mediated control of vesicular release. This is important because enhancing release probability via a local mechanism will allow PV-interneurons to function otherwise quite normally, with unaltered dendritic integration or AP firing. Rather, enhanced presynaptic release would serve as a pure gain-modifier of GABA signaling, increasing in response to glutamate or decreasing in response to GluN2D inhibition. Our data are consistent with the idea that PV-interneurons play a key role and support some aspects of the durable response, in that we can detect changes in plasticity that persist long after ketamine or NAB14 clearance. These data also support the idea that GluN2D inhibition could mimic the beneficial effects of ketamine without engaging its harmful actions, an idea also supported by a recent publication.^85^ In this respect, GluN2D inhibitors may provide an important new approach to treatment-resistant depression that is rapid-acting but does not carry many of the problems associated with ketamine use.

### Limitations of the study

One limitation of this study is that all of the electrophysiological and anatomical data that we obtained were from hippocampal and dentate neurons. Thus, it remains to be determined whether the roles we have established for presynaptic GluN2D-containing NMDARs will also be present in other brain regions. In addition, there is the potential for compensation in the parvalbumin-selective GluN2D knockout mice that could complicate the results obtained. For example, the increased locomotor activity we observed presented a confound for the behavioral assessment of amotivation. This prevented us from testing whether the results we obtained with GluN2C/D inhibitors and ketamine in our behavioral experiments were dependent on GluN2D in parvalbumin-interneurons, since increased locomotor activity could compromise the conclusions drawn from these mice.

## RESOURCE AVAILABILITY

### Lead contact

Further information and requests for resources and reagents should be directed to and will be fulfilled by the Lead Contact, Dr. Stephen Traynelis (strayne@emory.edu). Data reported in this paper will be shared by the lead contact upon request.

### Materials availability

- cDNAs and rAAV vectors generated in this paper are available upon request.
- SEP-GluN2D mice are available upon request.

### Data and code availability

- Data: Data reported in this paper will be shared by the lead contact upon request.
- Code: Code is available from GitHub at the following link https://github.com/MicrocircuitProjects/Presynaptic_GluN2D/releases/tag/Puncta_finder
- Other Items: Any additional information required to reanalyze the data reported in this paper is available from the lead contact upon request. All materials used are commercially available from indicated vendors; compounds synthesized (NAB-14, (S)-EU997–74) are commercially available from multiple vendors, and the synthetic methods are published. Software is commercially available from the indicated vendors.

## STAR★METHODS

### EXPERIMENTAL MODELS AND STUDY PARTICIPANTS

#### Animals

All procedures that involved the use of animals were approved by the Emory University, University of Colorado, and NICHD IACUCs. Sprague-Dawley rats were used to produce neuronal cultures. SEP-GluN2D C57VBl/6 mice were produced at Emory University as described in Figure S1. Mice used in this study were C57Bl/6J originally obtained from Jax. PV-GluN2D knockout mice were obtained by crossing PV-CRE (Jax 017320) and Flp conditional/Cre floxed GluN2D mice (B6Dnk;B6N-Grin2dtm1a(EUCOMM)Wtsi/H^86^), generously provided by Danny Winder. GluN2C knockout mice (RIKEN B6; 129S-Grin2c,tm1Nak>^88^) were generously provided by Stefano Vicini and Shigetada Nakanishi. PV-GluN2D knockout mice have been described by Gawande et al. (2023), and show no significant differences compared to PV-Cre controls in resting membrane potential, spike frequency, or action potential half-width, suggesting similar intrinsic properties between WT and PV-2D knockout PV interneurons. Our recordings (from the experiment in Figures 5I and 5J) show an identical IPSC time course for WT (tau 9.9 ± 3.8 ms, *n* = 6) and PV-GluN2D knockout mice (tau 10.5 ± 4.7 ms, *n*-7, *p* = 0.833). Breeder and offspring genotyping was done via Transnetyx for *Grin2d*-floxed and PV-Cre mice, *Grin2d* SEP knockin mice were genotyped by the Emory Mouse Transgenic and Gene Targeting Core by PCR followed by Sanger sequencing. All probes used are described in Table S4. Age is specified for all experiments. All experiments were performed on male and female mice except amotivation, which was performed on male mice (see below). The variability of the electrophysiological data did not allow assessment of sex differences.

#### Primary cultured neurons for immunocytochemistry and imaging

Dissociated hippocampal neurons were made from E19 male and female Sprague-Dawley rat embryos and plated at 75,000 cells per 24-well on #1.5 12-mm round coverslips coated with poly-D-lysine (Millipore). Neurons were maintained in NB/B27 medium (Thermo Fisher) and fed weekly by replacing half of the medium. Neurons were transduced with ALFA-mGluN2D lentiviral particles (40 μL per well) at days *in vitro* (DIV) 11 and processed for immunocytochemistry at DIV 23.

Dissociated hippocampal neurons were also prepared from male and female postnatal day 0–2 (P0–2) Sprague-Dawley rat pups. Rats were decapitated and the hippocampi were dissected out and dissociated using papain. Cells were plated at a density of 100,000 cells per well on 18-mm coverslips coated with poly-D-lysine (Sigma, P6407) and maintained in MEM with 10% FBS and penicillin/streptomycin in 12-well plates for 24h. The media was then exchanged for Neurobasal-A with B27 and Glutamax, and half of this media was exchanged again with fresh neurobasal A with B27, glutamax and mitotic inhibitors (uridine and fluoro deoxyuridine) at day 8 *in vitro* (DIV8). The neurons were cultured at 37°C in a humidified incubator with 5% CO_2_.

#### HEK 293 cell cultures and Xenopus laevis oocytes

HEK293T cells (CRL 1573, ATCC, Rockville, MD, hereafter HEK cells) were cultured in Dulbecco’s modified Eagle medium with GlutaMax-I (Invitrogen, Carlsbad, CA), 10% dialyzed fetal bovine serum, 10 U/mL penicillin, and 10 *μ*g/mL streptomycin at 37°C in an atmosphere of 5% CO_2_. HEK cells were authenticated by ATCC at the time of purchase. HEK cells were tested for mycoplasma contamination by means of a PCR-based assay and found to be negative. *Xenopus laevis* ovaries were purchased from Xenopus 1 (Dexter, MI).

### METHOD DETAILS

#### Chemicals

(S)-DQP997–74 was synthesized according to procedures described by D’Erasmo.^32^ (+)EU1180–453 was synthesized as described by Epplin.^31^ NAB14 was synthesized as described by Swanger.^55^ Each of these compounds was >95% pure as assessed by NMR and or LC-MS and made as a stock solution in DMSO at 100x-1000x the desired concentration; vehicle was always <0.1% DMSO. (+)-MK-801 maleate, gabazine, TFB-TBOA, QX-314, and NBQX were obtained from Cayman Chemicals. (−)-bicuculline methiodide and CNQX were obtained from Tocris, and D,L-AP5 sodium salt (D,L-2-amino-5-phosphonovalerate or APV) and 7-chlorokynurenate were obtained from Abcam. TTX and biocytin were obtained from Hello Bio. Anti-streptavidin Alexa Fluor 546 conjugate was obtained from ThermoFisher. All other chemicals were from Sigma-Aldrich.

#### NAB-14 formulation and *in vivo* pharmacokinetic study

NAB14 was formulated in 10% DMSO, 40% PEG400, and 50% sterile saline with gentle vortexing to make a stable, clear 1 mg/mL solution, made fresh prior to pharmacokinetic (PK) or behavior studies.

For the PK study, male and female C57Bl6 mice (14–23 weeks old) were dosed intraperitoneally (IP) in a 10 mL/kg injection volume. At the desired time point (0.25, 0.5, and 1 h post administration), each mouse was overdosed with isoflurane and decapitated with sharp scissors. Trunk blood was collected into K + EDTA sample tubes, stored on ice for 5–10 min, spun in a microfuge at 4°C for 10 min at 9500 rpm, then after a 100 μL plasma aliquot was removed and frozen on dry ice. Also, immediately after decapitation, a portion of the cerebral cortex was dissected from each mouse, weighed, and frozen on dry ice. Both plasma and brain samples were stored at −80°C until bioanalysis.

Plasma metabolites were extracted from 50 μL of plasma with the addition of 150 μL of 1 μg/mL of d_5_-ethoxycoumarin in MeCN followed by vortexing and then centrifugation at 14,000 rpm for 10 min at 4°C. Brain metabolites were extracted with the addition of 4X volume (μL) of 1 μg/mL d_5_-ethoxycoumarin in 70% MeCN/water per mg of brain sample, followed by homogenization with ceramic beads at 4°C for 3 cycles, and centrifugation at 14,000 rpm for 10 min at 4°C. The filtrate was then decanted into individual Eppendorf tubes and centrifuged again, after which the supernatant from each sample was transferred to autosampler vials for LC-MS/MS analysis.

#### Instrumentation and bioanalytical methods for pharmacokinetic data

Solutions containing 1 μg/mL d5-ethoxycoumarin internal standard in MeCN for plasma sample preparation and 70% MeCN/H_2_O for brain sample preparation were prepared fresh prior to sample preparation. A Biotage Lysera was utilized for the homogenization of brain samples. For the calibration curves, fresh intermediate stock solutions were prepared using serial dilutions from 10 μM of NAB14, with a further 100X dilution of each intermediate stock solution carried out in plasma or brain matrix, respectively. LC-MS/MS analysis was performed using an Agilent 1260 Infinity II HPLC system, which includes a micro vacuum degasser, quaternary pump, high-performance autosampler with thermostat, and a thermostatic column compartment coupled to an Agilent 6460C Triple Quadrupole Mass Spectrometer. The Agilent MassHunter Workstation (version B.02.00) was used for data acquisition, and MassHunter Quantitative analysis software (version B.01.04) was used for data analysis. The mass spectrometer operated in ESI mode with Agilent’s Jet Stream Technology. The Agilent MassHunter Optimizer software (B.02.00) was used to optimize the fragmentor voltage and collision energies. The optimizer also provided the most abundant multiple reaction monitoring (MRM) transitions necessary for detection. Methods were developed for NAB14 in the presence of an internal standard such as d_5_-ethoxycoumarin in positive mode. NAB14 was analyzed using MRM with quantifying and qualifying ions for increased reliability. Reverse-phase HPLC separation for each compound was achieved on an Agilent Poroshell Infinity 120-EC18 column (2.1 × 50 mm, 2.7 μm) with a mobile phase composed of acetonitrile (MeCN) in water spiked with formic acid (0.1%) at a flow rate of 0.5 mL/min. Column temperature was maintained at 40°C. Other MS conditions were as follows: dwell time 100 ms; gas flow 10 L/min; nebulizer pressure 45 psi; delta EMV 200 V.

All solvents for the LC-MS/MS analysis were HPLC grade. Methanol and water were purchased from Thermo Fisher, and acetonitrile was obtained from Sigma Aldrich. The HPLC-grade formic acid was obtained from Thermo Fisher. The LC-MS vials with embedded inserts with screw caps were obtained from VWR.

#### Biochemistry/Western blots

Western blot samples from homogenized male and female mouse brain were prepared in 4x Laemmli sample buffer and boiled at 95°C for 5 min. Samples were then run on mini-protean TGX stain-free protein gels (Bio-Rad) at 120V for about an hour. Gels were transferred onto PVDF membranes at 30V for 90 min. Blots were then blocked in 5% non-fat milk for 1 h with shaking at room temperature and incubated in primary antibodies overnight at 4°C while rocking. Blots were washed 3 times with TBS-T (TBS +1% Tween 20) for 10 min each with shaking. Blots were then incubated in secondary antibody for 1–2 h at room temperature with shaking, and were washed again 3 times in TBS-T for 10 min each before being developed using chemiluminescence (West Pico, Thermo Fisher). We used the following antibodies: rabbit anti-GFP (Abcam ab290), mouse anti-α-tubulin (Sigma T6199).

#### Stereotactic surgeries

10–12 week old male and female mice were injected with AAV.S5E2.tdTom (addgene 135630), AAV.S5E2.gCaMP6f (addgene 135632), AAV.S5E2.ChETA (addgene 220310), or AAV.S5E2.SEPTrak-GluN2D (our custom vector) in separate experiments. All AAV serotypes used were PHP.eB (Chan et al., 2017). When performing intracranial viral injections, mice were head-fixed in a stereotactic platform (RWD instruments) using mouse-fitting ear bars while under isoflurane anesthesia (1.8% inhalation). Thermoregulation was continuously provided by a heating plate using a rectal thermocouple for biofeedback to maintain core body temperature near 37°C throughout. Bupivacaine (0.75%) was subcutaneously injected into the scalp to induce local anesthesia. A small incision was opened 5 min thereafter, and a hole was drilled in the skull (<0.5 mm in diameter) to allow access for microinjection. Glass micropipettes for injection were fabricated using a vertical puller (Narishige). Coordinates (in mm from bregma) for microinjection were X = ± 2.02; Y = −2.46; α = 0°; Z = −1.55. Viral solution (titer 1 × 10^12^ to 1 × 10^13^ vg/mL) was injected slowly (~0.02 μL min-1) using a Picospritzer (0.3–0.5 μL total). After ejection of virus, the micropipette was held in place for ~5 min before slow withdrawal. The scalp was closed with surgical sutures followed by Vetbond tissue adhesive (3M), and the animal was allowed to recover under analgesia for 3 days thereafter. Animals were sacrificed 1–3 weeks later under full anesthesia for transcardial perfusions and preparation of acute slices.

#### Acute slice preparation

Coronal brain slices (250–300 μm) were made from 8 to 12 week old wild-type (WT), PV-GluN2D knockout, or GluN2C knockout mice, from both sexes for electrophysiology and 2-photon imaging. Mice were euthanized by isoflurane overdose followed by decapitation and removal of the brain. The brain was rapidly placed into ice-cold artificial cerebrospinal fluid (aCSF) composed of (in mM) 230 sucrose, 24 NaHCO_3_, 10 glucose, 2.5 KCl, 1.25 NaH_2_PO_4_, 3 Na-pyruvate, 5 Na-L-ascorbate, 12 N-acetyl-cysteine, 10 MgCl_2_, and 0.5 CaCl_2_ saturated with 95% O_2_/5% CO_2_. The brain was then glued to the removable stage and sectioned on a vibratome (Leica VT1200S, Wetzlar, Germany). After sectioning, slices were transferred to a holding chamber with the same solution, except that Mg^2+^ was lowered to 1.5 mM. Slices were incubated at 32°C for 30 min in this solution before returning them to room temperature for at least 1 h recovery prior to use.

#### Whole cell patch clamp recording from hippocampal slices

Slices were transferred to a submersion chamber and neurons were identified via IR-DIC on an upright microscope. Slices were continuously perfused at a rate of 4 mL/min with oxygenated standard recording aCSF containing (in mM) 126 NaCl, 26 NaHCO_3_, 10 glucose, 2.5 KCl, 1.25 NaH_2_PO_4_, 1.5 MgSO_4_, and 1.5 CaCl_2_ bubbled with 95% O_2_/5% CO_2_. The temperature of the external solution was heated to 30–32°C by a TC-344C inline heater system (Warner Instruments). The recording chamber was flushed with ~10 mL of 70% ethanol and ~20 mL of diH_2_O after each slice was recorded to ensure no residual drug remained. Recording electrodes were made from borosilicate glass (1.50 mm outer diameter, 1.12 mm inner diameter; World Precision Instruments) via micropipette puller (P-1000; Sutter Instrument); pipette resistances were 6–8 MΩ. Recordings were made using a Multiclamp 700B amplifier (Molecular Devices), filtered at 2 kHz using an 8-pole Bessel filter (−3dB), and digitized at 20 kHz (Digidata 1550B) using Axon pClamp10 software.

For intrinsic and action potential firing properties, the intracellular solution contained (in mM) 138 potassium gluconate, 4 MgCl_2_, 4 Na_2_ATP, 0.5 Na_2_GTP, 10 HEPES, 2 KCl, 3 L-ascorbic acid, and 0.5% biocytin. After obtaining a whole-cell configuration, cells were dialyzed for 5 min in current-clamp mode at I = 0. The liquid junction potential was not corrected, and all current-clamp responses were automatically bridge-balanced using the Multiclamp 700B clamp commander software. Input resistance was calculated by using the steady-state voltage change in response to a −10 pA 200 ms hyperpolarizing current injection period (Olah et al., 2024). Cellular electrical and firing parameters were established by recording voltage responses to current steps between −6 pA/pF (normalized to the cell’s capacitance in each recording measured immediately in voltage clamp after break-in) and 20 pA/pF or until the cell displayed depolarization-induced block of firing.

For voltage-clamp recordings of miniature inhibitory postsynaptic currents (mIPSCs) in 1 μM TTX, we utilized the two most common recording internals to ensure the reproducibility of our results. We recorded mIPSC frequency when holding near the reversal potential for EPSCs (+10 mV) to eliminate AMPA receptor mEPSCs with the electrodes filled with internal solution containing (in mM) 110 Cs-gluconate, 4 NaCl, 30 CsCl, 5 BAPTA, 5 HEPES, 0.5 CaCl_2_, 2 MgCl_2_, 2 Na-ATP, 0.3 Na-GTP. For some experiments, 50 μM MK-801 was added to the internal recording solution. We recorded mIPSCs in parallel experiments with an internal solution comprising 140 CsCl, 2 MgCl_2_ and 10 HEPES (Vm −50 mV^89^), and adding 10 μM CNQX or NBQX to the aCSF to block inward mEPSCs. The two conditions yielded indistinguishable results for mIPSC properties (see Figure S7), and thus data from these two conditions were pooled.

The experimental recording protocol was 5 min of baseline recording, followed by a wash-in of the test drug for 10 min. For some experiments, the recordings were finished by switching to aCSF that contained 10 μM gabazine to confirm that the currents we recorded were mediated by GABA_A_ receptors. For all cells tested, gabazine eliminated all miniature synaptic currents. The last 2 min of the control and first two minutes of drug application were used for data analysis. Evaluation of mIPSC frequency for recordings performed in aCSF (10 min) followed by vehicle (10 min) to mimic a typical 20 min experiment revealed no systematic shifts in frequency (averaged every 2.5 min, R^2^ = 0.195, *n* = 6 cells) over the course of an experiment, confirming that our recording protocol assessed stable mIPSC activity. mIPSCs were analyzed using MiniAnalysis (Synaptosoft).

Stimulus-evoked IPSCs were recorded from dentate granule cells (GC) using a stimulating electrode placed in close proximity to the GC body. Stimulation strength was set slightly above the minimal intensity needed to reliably evoke inhibitory events in every recording and to minimize failures. Any sweeps with failures were omitted from analysis. GC recording solution was 140 CsCl, 2 MgCl_2_ and 10 HEPES for mIPSC recordings, with added 2 mM QX-314 to block Na_v_ channels, and MK-801 (50 μM) in a subset of experiments as noted. Paired pulse ratio (PPR) was calculated from 2 stimuli elicited at either 50 or 100 ms inter-stimuli-intervals, delivered at 0.2 Hz. PPR in the main text was always at 50 ms inter-stimulus interval. PPR for 10 Hz inter-pulse intervals were 0.73 ± 0.032 vs. 0.67 ± 0.028 for control and PAM, respectively. For 20 Hz, PPR was 0.73 ± 0.028 vs. 0.63 ± 0.016 in control and with PAM, respectively. Measurements of the IPSC time course were performed on the first evoked event using only 100 ms inter-stimuli-interval experiments and fitting the decay of the current with an exponential function.

For optogenetic slice experiments, ChETA was excited using blue light from an unfiltered LED (M470L3; Thorlabs) centered on *l* = 461 nm (±20 nm). The LED was rapidly modulated with time-locked TTL pulses from the electrophysiology software using short pulses with a current controller (LEDD1B; Thorlabs). 5–10 ms pulses (respectively) were found to be optimal to reliably elicit IPSCs every trial with minimal jitter. To eliminate potential sources of variation between experiments, these parameters and the blue light power at the objective remained unaltered for all optogenetic control and test experiments.

Series resistance for mIPSCs was monitored throughout experiments by applying a 5 mV hyperpolarizing pulse every 30 s and was typically calculated to be in the range of 4–12 MΩ. For current clamp experiments, cells were briefly switched to voltage clamp and held at −60 mV while a series of 50 ms duration, 5 mV square hyperpolarizing potentials were applied to the cell. Current clamp recordings had series resistance measurements made at the beginning and at the end of each experiment, which usually lasted 5–10 min. All series resistances were measured offline by analyzing the peak of the capacitive charging spike and applying Ohm’s law. A cell was excluded if the series resistance (Rs) changed >25% during the experiment or exceeded 15 MΩ.

#### Field potential recordings

One day prior to hippocampal slice preparation, 4–14 week old mice (wild-type and PV-GluN2D knockout) of both sexes received a single 20 mg/kg i.p. injection of racemic ketamine (K2753, Millipore-Sigma) or vehicle (sterile saline). Alternatively, mice received an i.p. injection of 10 mg/kg NAB14 or vehicle (40% v/v PEG-400, 10% DMSO, 50% 5% dextrose in deionized water). 24 h later, mice were overdosed with isoflurane, the brain removed, and 400 μm coronal slices prepared as described above, except that the slices were kept in a holding chamber after sectioning for at least one hour at room temperature with aCSF, as described above, containing 1 mM CaCl_2_ and 2 mM MgSO_4_. The experimenter was blinded to pre-treatment with NAB14 or vehicle.

The protocol for recording and analyzing field potentials corresponding to stimulus-evoked field EPSPs within the *str. radiatum* close to the border of *str. pyramidale* was previously described.^90^ Hippocampal slices were transferred to an interface-style recording chamber (BSC 1–1, Scientific Systems Design, Inc, Canada) and bathed in recording aCSF described above with 3.0 mM CaCl_2_ and 1.5 mM MgSO_4_, heated to ~30°C. A concentric bipolar platinum-iridium electrode (#30200, FHC, ME, USA) was used to stimulate Schaffer collaterals at 0.05 Hz while a recording electrode was placed in the upper third of CA1 *str. radiatum*. Recording electrodes were made from borosilicate glass (1.50 mm outer diameter, 1.12 mm inner diameter; World Precision Instruments) by use of a micropipette puller (P-1000; Sutter Instrument); pipettes were filled with recording aCSF. Signals were obtained by A-M Systems field recording amplifier (Model 1800, A-M Systems, WA, USA) using 1,000x gain, with high and low pass filtering at 1 Hz and 5 kHz, respectively (−3 dB, Bessel). 60 cycle noise was removed using Hum Bug noise removers (Digitimer). Recordings were digitized at 20 kHz using NacGather acquisition software 2.07 (Theta Burst, Irvine, CA, USA).

Before each LTP recording, a brief input-output (I-O) curve was recorded with one sweep per stimulation level at 0–100 μA stimulus intensity, then the stimulation level was set to the level that yielded a response amplitude of ~50% compared to that obtained in response to 100 μA stimulation. In addition, a brief pair-pulse stimulation protocol (25 ms inter-pulse interval) was recorded to verify the proper location of the stimulation/recording sites. For PPR, there were no significant differences between vehicle and ketamine groups in either WT (vehicle 1.4 ± 0.05 *n* = 9, ketamine 1.4 ± 0.06, *n* = 9, *p* = 0.90 *t* test) or PV-2DKO (vehicle 1.4 ± 0.04 *n* = 13, ketamine 1.4 ± 0.04, *n* = 13, *p* = 0.5 *t* test). For PPR, there were no significant differences between vehicle and NAB14 groups (vehicle 1.4 ± 0.05 *n* = 27, NAB14 1.4 ± 0.03, *n* = 23, *p* = 0.94 *t* test). The LTP recording protocol consisted of 20-min low frequency stimulation at 0.05 Hz, followed by theta-burst stimulation (TBS, one train of 10 bursts comprised of four pulses at 100 Hz with an inter-burst interval of 200 ms), to induce an NMDAR-dependent form of long-term potentiation (LTP), followed by 1 h of low frequency stimulation at 0.05 Hz.^90,91^ Recordings were made from 2 slices simultaneously, with one slice from a vehicle-treated mouse and one slice from a ketamine-treated mouse. Unstable slices or slices not showing LTP were discarded.

#### Two-photon live imaging

Two-photon laser scanning microscopy was performed on hippocampal acute slices obtained form male and female mice (250 μm thick) continuously perfused with the same solution as for acute slice electrophysiology (except where noted) using a commercial scan head (Ultima; Bruker Corp) fitted with galvanometer mirrors (Cambridge Technology) using a 60x, 1.0 NA objective. The scan was incorporated into an Olympus upright microscope (BX51WI) using a 60X/1.0 NA water immersion objective (Olympus). Two-photon excitation was provided by a mode-locking Ti:sapphire laser (Chameleon Ultra II; Coherent). Fluorescence emission was detected using GaAsP photomultiplier modules (Hamamatsu) with a dichroic mirror and et640/120–2p and et510/80–2p bandpass filters (Chroma). For Ca^2+^ imaging, an analysis pipeline detected and tracked axonal puncta using scikit-image’s Difference of Gaussians blob detection algorithm, with phase cross-correlation for image registration to correct for drift between timepoints. Background-corrected fluorescence intensities were extracted for puncta in both control and drug treatment conditions, creating a master list for quantitative comparison. Only puncta that were automatically identified consistently throughout the entire experiment (i.e., not lost due to bleaching or otherwise) were included in our analyses. Trajectory analysis was performed using fractional changes ((F_t_ - F_0_)/F_0_), where F_0_ represents the average control intensity for all puncta (for 3 min before drug) and where F_t_ represents binned averages during drug treatment (25 s bins after local drug puffing began). All 2-photon imaging experiments were performed in ≥3 post-surgical mice for all conditions. Excitation wavelengths were set to optimize collection and chromatic separation (λ = 1040 nm for SEP/mScarlet experiments, with 910 nm for Ca^2+^ imaging). For Ca^2+^ imaging, image resolution was 512×512 and imaging frequency was 0.36 Hz.

#### Super-resolution imaging

Most super-resolution images were collected using the Nikon CSU-W1 SoRa Spinning Disk system in the SoRa4 microlensing configuration. The system is equipped with four laser lines (385 nm, 475 nm, 594 nm, and 621 nm). Oil immersion 60X (CFI60 PLAN APOCHROMAT LAMBDA D 60× Oil) objective was used for imaging and data was collected using a Hamamatsu ORCA-Fusion Gen III sCMOS camera. The XY/Z optical resolutions of the Sora system are 150 nm/320 nm and 120 nm/300 nm without and with deconvolution, respectively. Additionally, a Nikon SIM system with two lasers (488 nm and 560 nm) in our imaging core facility was also used for Structured Illumination Microscopy (SIM), which can achieve an optical resolution (XY/Z) of 115 nm/270 nm using 3-D SIM. An oil immersion 100X/NA1.49 Plan Apo objective was used for 3-D SIM imaging. Nikon Elements software was used for SIM and SoRa image acquisition and reconstruction.

#### Super-resolution image analysis

SoRa images were used to quantify the distribution of SEP puncta in different layers of the dentate gyrus. The analysis was carried out using a custom written Python script. Briefly, SoRa images with the same field of view, resolution and laser intensity were gathered from the granule cell layer, the inner and the outer molecular layer. These images were converted to NumPy arrays using the nd2 package. Puncta were identified using the blob_dog (Difference of Gaussians) algorithm of the scikit-image package, which approximates local peaks by blurring the image with increasing standard deviations and subtracting subsequent blurred samples. In this algorithm a putative lower and upper limit must be defined, which we set 1x and 4x of the x-y resolution of the system after deconvolution (120 nm lower limit and 480 nm upper limit). Furthermore, we allowed 20% overlap between two putative puncta. Image z-stacks were used for this analysis, but images were analyzed independently in the stack. The resulting 3d coordinates of the hits were merged into a 3-dimensional array and puncta which were present in at least 4 neighboring focal planes were accepted. The xy coordinates of the puncta were averaged from every focal plane the puncta were identified in, and Z-coordinates were assigned to the middle section. The analysis of Bassoon-SEP puncta difference was carried out using the same algorithm.

#### Immunohistochemistry

Male and female mice were overdosed with isoflurane, placed on their backs, and the chest cavity was opened. The diaphragm was immediately cut, and a 27 gauge needle was inserted into the left ventricle of the heart and the right ventricle cut to allow drainage. Mice were then perfused with PBS (7 mL/min) for 1 min followed by cold 4% paraformaldehyde (5 mL/min) for 5–8 min after which the brain was fixed in 4% PFA for 45 min at 4°C and either sectioned on a vibratome or placed in PBS solution at 4°C. Staining consisted of three steps, with 3–5 PBS wash cycles between each step. First, 50 μm thin slices were permeabilized for 20 min using 0.1% PBST and 0.3% Triton X-, followed by a 60-min blocking step with 0.1% PBST, 0.15% Triton X- and 5% normal goat serum (NGS). Next, primary antibodies, including chicken anti-GFP (Abcam, AB13970, 1:5000), rabbit anti-bassoon (Synaptic Systems, 141 003, 1:2000), and guinea pig anti-parvalbumin (Synaptic Systems, 195 308, 1:2000), were added to PBS, 0.15% Triton X-, and 5% NGS. Slices were incubated with antibodies at room temperature for 2 h, or at 4°C overnight. Finally, after 3–5 wash cycles, slices were developed using secondary antibodies (goat anti-guinea pig (Alexa Fluor 647, Abcam, AB150187 or Alexa Fluor 568, Abcam, AB175714, 1:500), goat anti-rabbit (Alexa Fluor 594, Invitrogen, A11012 or Alexa Fluor 647, Abcam, AB150079, 1:500), goat anti-chicken (Alexa Fluor 488, Invitrogen, A11039, 1:500)) at room temperature for 2 h, or at 4°C overnight. After a final wash cycle, slices were mounted on glass microscope slides and covered with Fluoromount-G with DAPI (Invitrogen).

#### Recordings from recombinant NMDA receptors expressed in HEK cells and oocytes

cDNAs encoding human GluN1 lacking exon5 (GluN1–1a; NCBI Reference Sequence GenBank: NM_007327.3) and GluN2D (GenBank: NM_000836.2) were synthesized, and SEP fused in-frame immediately after the signal peptide (Figure S1). For the synthesis of cRNA, cDNA constructs were first linearized using the appropriate restriction enzymes, purified using a QIAquick purification kit (Qiagen, Germantown MD), and then transcribed *in vitro* according to the manufacturer’s instructions (mMessage mMachine, Ambion, Austin, TX). *Xenopus laevis* oocyte preparation, cRNA synthesis, cRNA microinjections were all performed as previously described.^92,93^ Two-electrode voltage-clamp recordings were performed on oocytes expressing recombinant human GluN1/GluN2D or GluN1/GluN2D-SEP receptors at room temperature (23°C). Oocytes were injected with 5–10 ng of cRNA in RNAse-free water with a GluN1:GluN2 ratio of 1:10 and incubated at 15°C–19°C in Barth’s solution containing (in mM) 88 NaCl, 1 KCl, 2.4 NaHCO_3_, 10 HEPES, 0.82 MgSO_4_, 0.33 Ca(NO_3_)_2_, 0.41 CaCl_2_. The Barth’s solution was supplemented with 100 μg/mL gentamycin (Fisher BP918–1), 1 μg/mL streptomycin (Fisher 15140–12), and 1 U/mL penicillin (Fisher 15140–122). Approximately 2–4 days after injection, recordings were performed at room temperature using an extracellular recording solution containing (in mM) 90 NaCl, 1 KCl, 10 HEPES, 0.5 BaCl_2_, 0.01 EDTA and adjusted to pH 7.4 with NaOH. Agonist concentration–response curves were generated by applying a maximally effective concentration of glutamate (30 μM) and variable glycine, or a maximally effective concentration of glycine (30 μM) and variable glutamate. We assessed the IC_50_ value for Mg^2+^ inhibition of the response to maximally effective concentrations of glutamate (100 μM) and glycine (30 μM).

HEK cells were plated in 24-well plates on 5 mm glass coverslips (Warner Instruments, Hamden, CT) coated with 10–100 *μ*g/mL poly-D-lysine). Cells were transfected with plasmids encoding GluN1/GluN2D/GFP or GluN1/SEP-GluN2D/GFP at a mass ratio of 1:1:1 using the calcium phosphate method.^94^ The culture medium containing the transfection mixture was replaced after 4–6 h with DMEM supplemented with penicillin, streptomycin, and the NMDAR antagonists APV (200 *μ*M) and 7-chlorokynurenate (200 *μ*M). Experiments were performed 12–24 h post-transfection. Whole-cell voltage-clamp recordings were performed at V-_HOLD_ −60 mV at room temperature (23°C) using an Axopatch 200B amplifier (Molecular Devices, Union City, CA), low-pass filtered at 2–4 kHz (−3 dB, 8-pole Bessel; Frequency Devices, Haverhill, MD), and digitized at 40 kHz using Digidata 1322A or 1440A (Molecular Devices, Union City, CA) controlled by Clampex software (Molecular Devices, Union City, CA). Recording electrodes (3–4 MΩ) were pulled from thin-walled borosilicate glass (TW150F-4; World Precision Instruments, Sarasota, FL) and filled with (in mM) 110 D-gluconic acid, 110 CsOH, 30 CsCl, 5 HEPES, 4 NaCl, 0.5 CaCl_2_, 2 MgCl_2_, 5 BAPTA, 2 NaATP, and 0.3 NaGTP (pH 7.35 with CsOH). The extracellular solution contained (in mM) 150 NaCl, 10 HEPES, 3 KCl, 0.5 CaCl_2_, 0.01 EDTA, and 20 D-mannitol (adjusted to pH 7.4 with NaOH). Rapid solution exchange was accomplished through a two-barrel theta-glass controlled by a piezoelectric translator (Burleigh Instruments, Fishers, NY). Open tip junction currents using undiluted and diluted (1:2 in water) extracellular recording solution were used to estimate the speed of solution exchange at the tip, which had 10–90% rise times of 0.4–0.8 ms. Transfected HEK cells were patched and physically lifted from glass coverslips and placed in front of a two-barrel theta tube; the position (and flow pipe the cell was exposed to) was driven by a piezoelectric translator. To start, all cells were continuously bathed in saturating glycine (100 μM) and then rapidly exposed to saturating concentrations of both glutamate (1 mM) and glycine (100 μM) for 10 ms or 1 s, then moved back into saturating glycine (100 μM).

#### Cloning of ALFA-GluN2D and Production of Lentivirus particles

The sequence encoding the ALFA epitope tag (SRLEEELRRRLTE; Goetzke et al., 2019), flanked on both sides by proline residues, was inserted into mouse GluN2D (GenBank: NM_0081723.3) downstream of the sequence encoding the signal peptide. The lentiviral expression vector pSMPUW(hSynI) was derived from promoterless vector pSMPUW (Cell Biolabs) by insertion of the human Synapsin I promoter sequence followed by the β globin intron. The ALFA-mGluN2D open reading frame was then transferred from shuttle vector pUCIDT-Amp (IDT DNA technologies) to pSMPUW(hSynI) by InFusion assembly (Takara).

Lentiviral particles harboring the hSynI_ALFA-mGluN2D transcription unit were made in the 293-LTV cells line (Cell Biolabs). In brief, 293-LTV cells were plated on two 150 mm round culture dishes and grown until sub-confluent (70–80%). Cells were then transfected with transfer plasmid pSMPUW(hSynI)_ALFA-mGluN2D, envelope plasmid pMD2.G (Addgene #12259), packaging plasmid psPAX2 (Addgene #12260) and translational enhancer plasmid pAdVAntage (Promega) at a mass ratio of 4:2:2:1, using LipoD293 transfection reagent (SignaGen Laboratories). Culture supernatants were harvested at 48 h and 72 h, and viral particles were subsequently concentrated using Lenti-X concentrator (Takara). Viral pellets were reconstituted in 1 mL Neurobasal/B27 (NB/B27) medium (Thermo Fisher) and stored in small aliquots at −80°C.

#### Immunocytochemistry and imaging of transduced cultured neurons

Lentivirus-transduced neurons (see Experimental Models above) were live-labeled for 30 min at 37°C using Cy3-conjugated single-domain anti-ALFA nanobody (FluoTag-X2; NanoTag Biotechnologies) diluted 1:200 in growth medium (25 nM final concentration). Unbound antibody was removed by three brief washes with prewarmed NB/B27 medium. Neurons were then fixed and processed for post-fixation antibody labeling. Neurons were fixed for 15 min at room temperature in 4% paraformaldehyde, 4% sucrose in PBS, washed in PBS and then incubated for 30–60 min in 10% normal donkey serum (NDS), 0.1% Triton X-100 in PBS. Neurons were incubated with primary antibodies diluted in PBS containing 2% NDS and 0.1% Triton X-100 for 1–2 h at room temperature or overnight at 4°C. Primary antibodies were: rabbit polyclonal anti-GluA1 (raised against aa271–285 of rat GluA1; 10 μg/mL [unpublished]); guinea pig polyclonal anti-VGLUT1 (Synaptic Systems #135304; 1:2,000). Coverslips were washed in PBS and then incubated in secondary antibodies (Jackson Immunoresearch) diluted 1:1,000 in PBS containing 2% NDS and 0.1% Triton X-100 for 1 h at room temperature. Coverslips were washed again in PBS, dipped in water and mounted on glass slides in Mowiol/DABCO mounting medium. Neurons were imaged on a Zeiss LSM 900 confocal microscope at 63× magnification. Image stacks covering the entire depth of the dendritic arbor were acquired and projected in Z using the maximum intensity method. Images were adjusted for brightness in ImageJ/FIJI.

#### Live imaging of transfected cultured neurons

Neurons (see Experimental Models above) were transfected at DIV18 using Lipofectamine 2000 and imaged at DIV21. The SEPTrak constructs used encode an extracellular SEP-tag, the PDGFR transmembrane domain from the pDisplay vector, and an intracellular mScarlet fluorophore, either alone (control) or followed by the C-terminal tail of GluN2D. Prior to imaging, we enhanced the SEP signal in neurons by surface-labelling with Alexa 647-conjugated anti-GFP nanobodies (FluoTag-X4 anti-GFP, NanoTag Biotechnologies) diluted in culture media for 20 min. Neurons were imaged live at 32°C in aCSF (130 mM NaCl, 5 mM KCl, 10 mM HEPES, 30 mM glucose, 1 mM MgCl_2_, 2 mM CaCl_2_) on an Olympus IX71 equipped with a spinning-disc confocal scan head (Yokogawa). Excitation illumination was delivered from an acousto-optic tunable filter (AOTF) controlled laser launch (Andor). Images were acquired using 60X Plan-Apochromat 1.4 numerical aperture (NA) or 40X UApo/340 1.35 NA objectives and collected on a 1,024 × 1,024 pixel Andor iXon EM-CCD camera. z stack images were acquired using Metamorph software, and later analyzed as max-projections in ImageJ. To capture a large field of view at high resolution, multiple overlapping images of neuronal somata, axons and dendrites were captured and stitched together using the Grid/Collection stitching plugin in ImageJ. Axons were outlined manually using the polygon selection tool, and clusters of nanobody-labelled SEP were identified using the Analyze particles function with the threshold set 5 times higher than the background-subtracted mean intensity along the entirety of the axonal region to be analyzed. Cluster density was then calculated as the number of clusters identified divided by the length of the axon analyzed in micrometers.

#### Corticosteroid exposure and assessment of motivated behavior

Corticosterone hemisuccinate (4-pregnen-11β 21-diol-3 20-dione 21-hemisuccinate) (Steraloids, Inc.) was dissolved in drinking water by stirring overnight and used within 72 h of preparation. Male mice (P60-P165) were given the corticosterone solution (25 μg/mL free-base) in place of normal drinking water for 14 days. Mice were then weaned as follows: 3 days with 50% of the original CORT concentration, 3 days with 25%, followed by regular water, to allow for recovery of endogenous corticosterone secretion.^61,62^ Mice were males to avoid sex differences in corticosteroid-induced depression-like behavior.

Next, mice were food-restricted to not less than 90% of their free-feeding body weight to motivate food-reinforced responding. Testing was conducted using operant conditioning chambers equipped with two nose-poke apertures (left vs. right) and a separate magazine for food delivery (Med Associates). Mice were trained to nose poke for 20 mg grain and chocolate pellets (Bio-Serv) using a fixed ratio 1 (FR1) schedule of reinforcement, whereby each nose-poke resulted in the delivery of a single reinforcer. Mice underwent daily training sessions lasting 70 min or until mice earned 30 reinforcers from responding on each aperture (60 reinforcers/session). Mice were then reinforced on the nose-poke aperture that they favored during training according to a progressive ratio schedule of reinforcement, such that each reinforcer required the mice to increase responding by 4 (i.e., the first pellet was given after a single poke, the next required 5 pokes, the next required 9 pokes, and so forth). Sessions lasted either 180 min or until mice failed to respond for 5 min. Breakpoint ratios were defined as the highest response requirement completed during the session.

In the progressive ratio task, low break points following corticosterone models amotivation in depression.^61,62^ Consistent with this notion, chronic treatment with traditional antidepressants recovers break point ratios.^61,62^ Here, vehicle (40% v/v PEG-400, 10% DMSO, 50% 5% dextrose in deionized water) or NAB14 or ketamine (both 10 mg/kg, i.p., dissolved in vehicle and delivered at 1 mL/100 g) was administered. Mice were tested 24 h following injections, aligning with the durable physiological consequences of these drugs. Moreover, mice were tested in two conditions, with the drugs paired with reward delivery (“paired”) or when they were simply injected and left undisturbed for 24 h (“unpaired”). The purpose of these two testing conditions was to ascertain whether the antidepressant-like properties of acute NAB14 or ketamine were dependent on being paired with the behavioral testing procedure (akin to an adjunct therapy) or, instead, could be delivered independently of behavioral testing, having lasting consequences. Break points were compared across groups and between conditions.

Given the largely unknown behavioral consequences of NAB14, we also confirmed that it had no effects on low-effort responding (one poke results in one food pellet) by administering NAB14 or vehicle in a counter-balanced order to mice that had been trained to respond for food reinforcers as described above. Mice were placed in the chambers 30 min following injection. Responses/min with and without drug were compared.

#### Assessment of locomotor activity

Behavior assessments of locomotor activity were conducted by Emory University Rodent Behavioral Core (RRID:SCR_012499). Adult mice (P50–90, equal numbers of males and females) mice were habituated in the recording room for 48–72 h prior to the recording. Mice were first put into the locomotion recording chamber to do a 120-min baseline locomotion recording. Then the freshly made ketamine (10 mg/kg) or NAB-14 (10 mg/kg) or vehicle (40% v/v PEG-400, 10% DMSO, 50% 5% dextrose in deionized water) was i.p. injected with the injecting volume of 10 mL/kg. After injection, mice were put back into the same recording chamber for 60-min post-injection locomotion recording. Locomotion was recorded by Photobeam Activity System (San Diego Instruments), and the locomotion data was acquired as total ambulation distance (in centimeters) for every 5 min.

### QUANTIFICATION AND STATISTICAL ANALYSIS

Whole-cell electrophysiology data were collected by Clampex 10.7 with MultiClamp 700B Commander. mIPSC data were analyzed offline using MiniAnalysis 6 (Synaptosoft). MiniAnalysis used an 8-pA amplitude threshold for event detection, which was 5-fold higher than our peak RMS noise. For all whole-cell current responses, the average response per condition was analyzed offline using custom software. Rise time was measured as the 10–90% fractional response of the rising phase of each current response. Peak amplitude was determined by finding the maximum of a moving search average of 3–5 data points. The deactivation time course for evoked NMDAR-mediated whole-cell currents was measured by fitting a dual-exponential function to the decay; the time constants are reported as weighted deactivation time constants (τ_W_), calculated using the following equation:

$$\tau_{\text{W}}=\left[{\text{AMP}}_{\text{FAST}}/\left({\text{AMP}}_{\text{FAST}}+{\text{AMP}}_{\text{SLOW}}\right)\right]\tau_{\text{FAST}}+\left[{\text{AMP}}_{\text{SLOW}}/\left({\text{AMP}}_{\text{FAST}}+{\text{AMP}}_{\text{SLOW}}\right)\right]\tau_{\text{SLOW}}$$

Field recording data was analyzed in NACShow (v. 2.0.2.6, Theta Burst). Amplitude and falling phase slope (10–90%) analyses yielded identical results; the slope is shown. Values from each slice were normalized to the average of 1 min before LTP induction and then averaged across all slices.

Custom Python code was written to analyze super-resolution images. Briefly, 2d arrays of each imaging channel were processed using the blob_dog algorithm of the scikit-image package. This algorithm relies on the Difference of Gaussian method to find the coordinates and radius of 2D circular or punctate objects in a gray-scaled image. The coordinates were decimated by accounting for the Z point spread function of the imaging setup, and the highest intensity points around the Z-spread were determined as the puncta Z center. Coordinates and fluorescence intensities in different channels were collected.

Data analyses and figures were made with GraphPad Prism 9.3.1 (GraphPad Software, LLC) and Illustrator (Adobe). Data are presented as mean ± SEM. Significance was determined via Student’s *t* test or ANOVA with repeated measures as appropriate. Post-hoc comparisons of break points in behavioral tests were made using Fisher’s LSD tests. An α value of *p* < 0.05 was considered statistically significant. When multiple tests were performed on the same recording, α values were corrected to prevent family-wise errors using the Bonferroni post-hoc correction method. All studies were designed so that an effect size of at least 1 was detected at 80% or greater power.

## Supplementary Material

1

Supplemental information can be found online at https://doi.org/10.1016/j.celrep.2026.117707.

## Figures and Tables

**Table:** KEY RESOURCES TABLE

REAGENT or RESOURCE	SOURCE	IDENTIFIER
Antibodies		
Rabbit anti-GFP	Abcam	AB290; RRID: AB_303395
Mouse anti- α-tubulin	Sigma	T6199; RRID: AB_477583
Chicken anti-GFP	Abcam	AB13970; RRID: AB_300798
Rabbit anti-Bassoon	Synaptic Systems	141003; RRID: AB_887697
Guinea pig anti-parvalbumin	Synaptic Systems	195308; RRID: AB_2927389
Goat anti-guinea pig Alexa Fluor 647	Abcam	AB150187; RRID: AB_2827756
Goat anti-guinea pig Alexa Fluor 568	Abcam	AB175714; RRID: 2864763
goat anti-rabbit Alexa Fluor 594	Invitrogen	A11012; RRID: AB2534079
goat anti-rabbit Alexa Fluor 647	Abcam	AB150079; RRID: AB_2722623
goat anti-chicken Alexa Fluor 488	Invitrogen	A11039: RRID: AB_2534096
Cy3-conjugated single-domain anti-ALFA nanobody (FluoTag-X2; NanoTag Biotechnologies)	NanoTag Biotechnologies	N1502
rabbit polyclonal anti-GluA1 (raised against aa271-285 of rat GluA1; 10 μg/mL)	In-house (Buonanno Lab)	N/A
guinea pig polyclonal anti-VGLUT1	Synaptic Systems	#135304; RRID: AB_887878
Alexa 647-conjugated anti-GFP nanobodies (FluoTag^®^-X4 anti-GFP)	NanoTag Biotechnologies	N0304; AB_2905517
Donkey anti-rabbit Alexa 488	Jackson Immunoresearch	Cat-# 711-545-152; AB_2313584
Donkey anti-guinea pig Alexa 647	Jackson Immunoresearch	Cat-# 706-605-148; AB_2340476
Bacterial and virus strains		
AAV.S5E2.tdTom	Addgene	Addgene 135630
AAV.S5E2.gCaMP6f	Addgene	Addgene 135632
AAV.S5E2.ChETA	Addgene	Addgene 220310
AAV.S5E2.SEPTrak-GluN2D	This paper	N/A
hSynI_ALFA-mGluN2D Lentivirus	This paper	N/A
Chemicals, peptides, and recombinant proteins		
(S)-DQP997-74	D’Erasmo et al.^32^	N/A
(+)EU1180-453	Epplin et al.^31^	N/A
NAB14	Swanger et al.^55^	N/A
(+)-MK-801 maleate	Cayman	Cat#10009019-50; CAS# 77086-22-7
gabazine	Cayman	Cat# 14585-50; CAS# 104104-50-9
(−)-bicuculline methiodide	Tocris/R&DSystems	Cat# 2503/50; CAS# 40709-69-1
CNQX	Tocris/Fisher/R&D Systems	Cat#1045/10; CAS#479347-85-8
NBQX	Cayman	Cat#14914-50; CAS#479347-86-9
D,L-AP5	Abcam	Cat# ab120271-50; CAS# 1303993-72-7
TTX	Hello Bio	Cat# HB1035-1; CAS# 18660-81-6
Biocytin	Hello Bio	HB5035; CAS# 576-19-2
TFB-TBOA	Cayman	Cat#31417-10; CAS#
Ketamine	Millipore-Sigma	K2753
QX-314	Cayman	10011032-50
7-chlorokynurenate	Abcam	Ab120255-50mg
Streptavidin Alexa Fluor 546 conjugate	Invitrogen	S11225
Corticosterone hemisuccinate	Steraloids	Q1562-000; CAS# 10215-77-7
Critical commercial assays		
Transnetyx automated genotyping	Transnetyx	https://quickorder.transnetyx.com
Experimental models: Cell lines		
HEK-293	ATCC	CRL-1573
Experimental models: Organisms/strains		
Mouse: Grin2D SEP KI	This paper	N/A
Mouse: B6.129P2-*Pvalb*^*tm1(cre)Arbr*^/J	The Jackson Laboratory	Jax 017320; RRID:IMSR_JAX:017320
Mouse: B6Dnk;B6N-Grin2dtm1a(EUCOMM)Wtsi/H	Salimando et al.^86^	N/A
Mouse: B6;129S(Cg)-Grin2c<tm1Nak>	RIKEN	RBRC02258
Mouse: C57Bl6	The Jakson Laboratory	Jax: 000664, RRID:IMSR_JAX:000664
Oligonucleotides		
Primers for PCR genotyping, see Table S4	This paper	N/A
Recombinant DNA		
pcIneo-human GluN1	This paper	GenBank: NM_007327.3
pcIneo-human GluN2D	This paper	GenBank: NM_000836.2
pcIneo-SEP-human GluN2D	This paper	N/A
pTrak	This paper	N/A
pTrak-GLuN2D-Cterminal	This paper	N/A
Software and algorithms		
ImageJ	Schroeder et al.^87^	https://imagej.net/ij/
MiniAnalysis 6	Synaptosoft	N/A
Automated SEP Puncta Analysis	This paper	https://github.com/MicrocircuitProjects/Presynaptic_GluN2D 10.5281/zenodo.20329522
MassHunter Quantiative Analysis	Agilent	https://www.agilent.com/en/promotions/masshunter-mass-spec?srsltid=AfmBOopV56bG9iPOC0TYtTktgMQ_aaf-YSWZFmW-G2q0IfQ4ll-rRBw
Axon pClamp 10 (Clampex)	Molecular Devices	https://www.moleculardevices.com/products/axon-patch-clamp-system/acquisition-and-analysis-software/pclamp-software-suite
NacGather Acquisition	Theta Burst Corporation	N/A - abandonware
Nikon Elements	Nikon	https://www.microscope.healthcare.nikon.com/products/software/nis-elements
Metamorph	BioVision Technologies	https://www.biovis.com/metamorph.html
Adobe Illustrator	Adobe	https://www.adobe.com/products/illustrator/
GraphPad Prism	GraphPad	https://www.graphpad.com/

## Highlights

- GluN2D-NMDARs cluster at PV-interneuron presynaptic boutons in the hippocampus
- GluN2D senses ambient glutamate to gate stochastic and evoked GABA release
- Ketamine’s rapid and durable effects on inhibition and LTP require GluN2D-NMDARs in PV cells
- Ketamine and GluN2C/D inhibition both ameliorate amotivation following chronic corticosterone
