# Supplementary material for: Bidirectional control of vesicular GABA release, LTP, and amotivation by GluN2D-selective allosteric modulators and ketamine
Source: Cell Rep. Author manuscript; Available in PMC 2026 Sep 16. (PMC13581442; doi:10.1016/j.celrep.2026.117707)
Supplement: Supplementary file 1 [file NIHMS2206882-supplement-1.pdf]

Cell Reports, Volume *45*

Supplemental information

Bidirectional control of vesicular GABA release, LTP, and amotivation by GluN2D-selective allosteric modulators and ketamine

Viktor J. Oláh, Isabelle F. Witteveen, Tue G. Banke, Alexa M. Leahy, Ryan Keable, Riley E. Perszyk, Cara T. Khayat, TrangKimberly T. Nguyen, Brianna J. Bixler, Frances G. Ghinger, Detlef Vullhorst, Yeeun Yook, Scott J. Myers, Elijah Z. Ullman, Jing Zhang, Hao Xing, John F. Traynelis, Eva S. Diaz, Sukhan Kim, Sophia Boraschi, Nicholas S. Akins, Ken H. Liu, Alet van der Westhuyzen, Yanli Yang, Chad R. Camp, Andres Buonanno, Randy A. Hall, James Q. Zheng, Dennis C. Liotta, Matthew J. Kennedy, Katherine W. Roche, Shannon L. Gourley, Hongjie Yuan, Matthew J.M. Rowan, and Stephen F. Traynelis

Supplemental Table S1: mIPSC Frequency Analysis

Genotype	Brain Region	Test Compound	Freq. Control (mean ± SEM)	Freq. Drug (mean ± SEM)	Freq. Fold (mean ± SEM)	N	p
**WT**	CA1	PAM	1.71 ± 0.22	2.38 ± 0.38	1.41 ± 0.21	14	0.0349
**WT**	DG	PAM	1.02 ± 0.20	1.18 ± 0.25	1.17 ± 0.05	12	0.0336
**WT**	CA1	NAM	0.91 ± 0.23	0.70 ± 0.18	0.77 ± 0.08	14	0.0175
**WT**	DG	NAM	0.66 ± 0.11	0.38 ± 0.06	0.57 ± 0.07	10	0.0039
**PV-2DKO**	CA1	PAM	1.34 ± 0.17	1.42 ± 0.25	1.04 ± 0.08	10	0.5431
**PV-2DKO**	DG	PAM	0.93 ± 0.21	0.93 ± 0.22	0.97 ± 0.08	9	0.8651
**PV-2DKO**	CA1	NAM	0.60 ± 0.13	0.67 ± 0.13	1.15 ± 0.08	9	0.1028
**PV-2DKO**	DG	NAM	0.68 ± 0.17	0.63 ± 0.14	0.96 ± 0.03	7	0.1993
**2CKO**	CA1	PAM	1.44 ± 0.24	1.99 ± 0.37	1.36 ± 0.12	9	0.0102
**2CKO**	DG	PAM	0.59 ± 0.09	0.82 ± 0.13	1.40 ± 0.10	10	0.0053
**2CKO**	CA1	NAM	2.07 ± 0.45	1.35 ± 0.37	0.63 ± 0.11	7	0.0287
**2CKO**	DG	NAM	0.72 ± 0.13	0.47 ± 0.08	0.69 ± 0.08	8	0.0122
**WT**	CA1	KET	1.40 ± 0.22	0.89 ± 0.19	0.60 ± 0.09	10	0.0024
**PV-2DKO**	CA1	KET	1.24 ± 0.38	1.11 ± 0.34	0.94 ± 0.06	9	0.1678
**WT**	CA1	TBOA	1.32 ± 0.17	1.58 ± 0.23	1.25 ± 0.11	14	0.0402
**WT**	CA1	Vehicle	1.85 ± 0.37	1.93 ± 0.42	1.02 ± 0.06	10	0.4594
**WT**	CA1	APV+TBOA	1.17 ± 0.37	1.03 ± 0.27	1.08 ± 0.13	13	0.5643
**PV-2DKO**	CA1	TBOA	0.82 ± 0.15	0.88 ± 0.18	1.14 ± 0.14	11	0.4897
**WT**	CA1	NAB	0.79 ± 0.12	0.61 ± 0.13	0.78 ± 0.12	7	0.0469
**WT**	CA1	MK801+PAM	1.22 ± 0.43	1.48 ± 0.48	1.44 ± 0.20	10	0.0140

Frequency is given in Hz. Freq fold is the frequency in drug divided by frequency in baseline. N is the number of cells. P was determined by paired t-test. PAM was 10 μM (+)EU1180-453, NAM was 1 μM (S)-EU997-74, KET was 1 μM ketamine, TBOA was 1 μM TFB-TBOA, APV was 400 μM D,L-AP5, MK801 was 50 μM MK-801.

Supplemental Table S2: Amplitude analysis

Genotype	Brain Region	Test Compound	Amp. Control (mean ± SEM)	Amp. Drug (mean ± SEM)	Amp. Fold (mean ± SEM)	N	p
**WT**	CA1	PAM	26.08 ± 3.68	27.45 ± 3.74	1.06 ± 0.04	14	0.1706
**WT**	DG	PAM	26.81 ± 4.50	26.73 ± 4.75	1.00 ± 0.05	12	0.9443
**WT**	CA1	NAM	23.53 ± 2.18	23.99 ± 2.31	1.04 ± 0.07	14	0.7216
**WT**	DG	NAM	35.95 ± 4.77	33.60 ± 4.27	0.94 ± 0.06	10	0.3014
**PV-2DKO**	CA1	PAM	16.08 ± 2.47	15.22 ± 2.50	0.94 ± 0.05	10	0.2123
**PV-2DKO**	DG	PAM	37.30 ± 3.25	37.61 ± 3.32	1.01 ± 0.05	9	0.8441
**PV-2DKO**	CA1	NAM	11.84 ± 0.90	12.55 ± 1.12	1.06 ± 0.05	9	0.2396
**PV-2DKO**	DG	NAM	33.95 ± 3.28	34.16 ± 3.23	1.01 ± 0.03	7	0.8303
**2CKO**	CA1	PAM	17.30 ± 3.02	17.16 ± 2.97	1.03 ± 0.05	9	0.5956
**2CKO**	DG	PAM	21.58 ± 2.26	20.63 ± 2.21	0.97 ± 0.05	10	0.4680
**2CKO**	CA1	NAM	23.41 ± 5.35	23.90 ± 6.51	1.03 ± 0.10	7	0.8464
**2CKO**	DG	NAM	23.37 ± 5.79	25.24 ± 6.19	1.13 ± 0.08	8	0.2294
**WT**	CA1	KET	15.07 ± 1.72	14.88 ± 1.65	0.99 ± 0.03	10	0.6154
**PV-2DKO**	CA1	KET	16.84 ± 1.91	17.33 ± 2.22	1.03 ± 0.03	9	0.4717
**WT**	CA1	TBOA	25.03 ± 1.48	24.62 ± 1.62	0.98 ± 0.03	14	0.6244
**WT**	CA1	Vehicle	21.16 ± 1.91	20.04 ± 1.58	0.96 ± 0.04	10	0.2087
**WT**	CA1	APV+TBOA	31.08 ± 2.06	30.34 ± 1.87	0.98 ± 0.03	13	0.4631
**PV-2DKO**	CA1	TBOA	20.12 ± 1.14	18.61 ± 1.02	0.94 ± 0.04	11	0.0882
**WT**	CA1	NAB	14.98 ± 1.53	14.11 ± 1.13	0.96 ± 0.07	7	0.4616
**WT**	CA1	MK801+PAM	14.02 ± 1.54	13.68 ± 1.24	1.00 ± 0.05	10	0.6211

Amplitude is given in pA. Amp. Fold is the amplitude in drug divided by amplitude in baseline. N is the number of cells. P was determined by paired t-test. PAM was 10 μM (+)EU1180-453, NAM was 1 μM (S)-EU997-74, KET was 1 μM ketamine, TBOA was 1 μM TFB-TBOA, APV was 400 μM D,L-AP5, MK801 was 50 μM MK-801.

Supplemental Table S3: NAB14 PK following 10 mg/kg i.p. delivery in adult C57Bl/6 mice

	Time (hr)	ng/ml (mean ± STD)	μM	N (mice)	Blood/Plasma ratio
Plasma	0.25	2362 ± 526	6.7	3	--
Plasma	0.5	1777 ± 54	5.1	3	--
Plasma	1	636	1.8	2	--
Brain	0.25	2683 ± 617	7.6	3	1.14
Brain	0.5	2292 ± 99	6.5	3	1.29
Brain	1	859	2.4	2	1.35

Supplemental Table S4: PCR Primers

Name	PCR primers
Grin2d-floxed	Forward primer GCTGGCGCCGGAAC
	Reverse primer GCGACTATAGAGATATCAACCACTTTGT
	Reporter AAGCTGGGTCTAGATATC
Grin2d-wildtype	Forward primer GGTCCCAGAGAGAAGACTATGCTT
	Reverse primer GGCCTGTCTTCCCCATCTC
	Reporter ATGGCCAGCAAAGAAA
PV-Cre	Forward primer CTAATTCCATCAGAAGCTGGTACCTT
	Reverse primer GGAGGGCCGCGAGAA
	Reporter ACCCAAGCAGAAGCT
PV-wildtype	Forward primer CACTCTGGTGGCTGAAAGCTAA
	Reverse primer GGAGGGCCGCGAGAAG
	Reporter CAAGCAGTCAGCGCCAC
Grin2d SEP KI	5’ Forward Primer CCTTATCTCTGTCCCTCATCTCTG
	5’ Reverse Primer TGACAAGTGTTGGCCATGGAACAG
	3’ Forward Primer GATGGAGGCGTTCAACTAGCAGAC
	3’ Reverse Primer CAGGAAGTCGAGAATGGGCGCGAC
	5’ Positive Control Forward Primer CCTTATCTCTGTCCCTCATCTCTG
	3’ Positive Control Reverse Primer CAGGAAGTCGAGAATGGGCGCGAC

A

Long ssDNA Donor- 1111 bp

GCCCCTCCCTCTCTCCATCACCCAACCTTCCCCTCTCGCCCCGTCTCTCCCGTGTCTCCCCTCGGGCTCTGCCCTCGCCTCCCGTCCACGC
AGAGGCCGCCCGGCGGGGCCCGCAGGCGATGCGCGGCGCCGGTGGCCCCCGCGGCCCTCGGGGCCCCGCTAAGATGCTGTTGCTG
CTGGCGCTGGCGTGCGCCAGCCCGGGGcgcgccagtaaaggagaagaacttttcactggagttgtcccaattcttgttgaattagatggtgatgttaatgggcac
aaattttctgtcagtggagagggtgaaggtgatgcaacatacggaaaacttacccttaaatttatttgcactactggaaaactacctgttccatggccaacacttgtcactactct
gacctatggtgttcaatgcttttcaagatacccagatcatatgaaacggcatgactttttcaagagtgccatgcccgaaggttatgtacaggaaagaactatatttttcaaagat
gacgggaactacaagacacgtgctgaagtcaagtttgaaggtgatacccttgttaatagaatcgagttaaaaggtattgattttaaagaagatggaaacattcttggacacaa
attggaatacaactataacgatcaccaggtgtacatcatggcagacaaacaaaagaatggaatcaaagctaacttcaaaattagacacaacattgaagatggaggcgttca
actagcagaccattatcaacaaaatactccaattggcgatggcccgtccttttaccagacaaccattacctgtttacaacttctactctttcgaaagatcccaacgaaaagag
agaccacatggtccttcttgagtttgtaacagctgctgggattacacatggcatggatgaataccTTAAGTTCCCGGAGGAGGTGCCGGGGCCGGGCGCGGC
CGGCGGGGGCACGGGCGGGGCGCGGCCGCTCAACGTGGCGCTGGTCTTCTCTGGCCCGGCGTACGCGGCCGAGGCGGCGCGCTTG
GGCCCGGCCGTGGCGGCGGCAGTGCGCAGCCCGGGCCTGGACGTACGGCCCGTGGCGCTGGTGCTCAACGGCTC

Signal Peptide
SEP
Exon 1

B

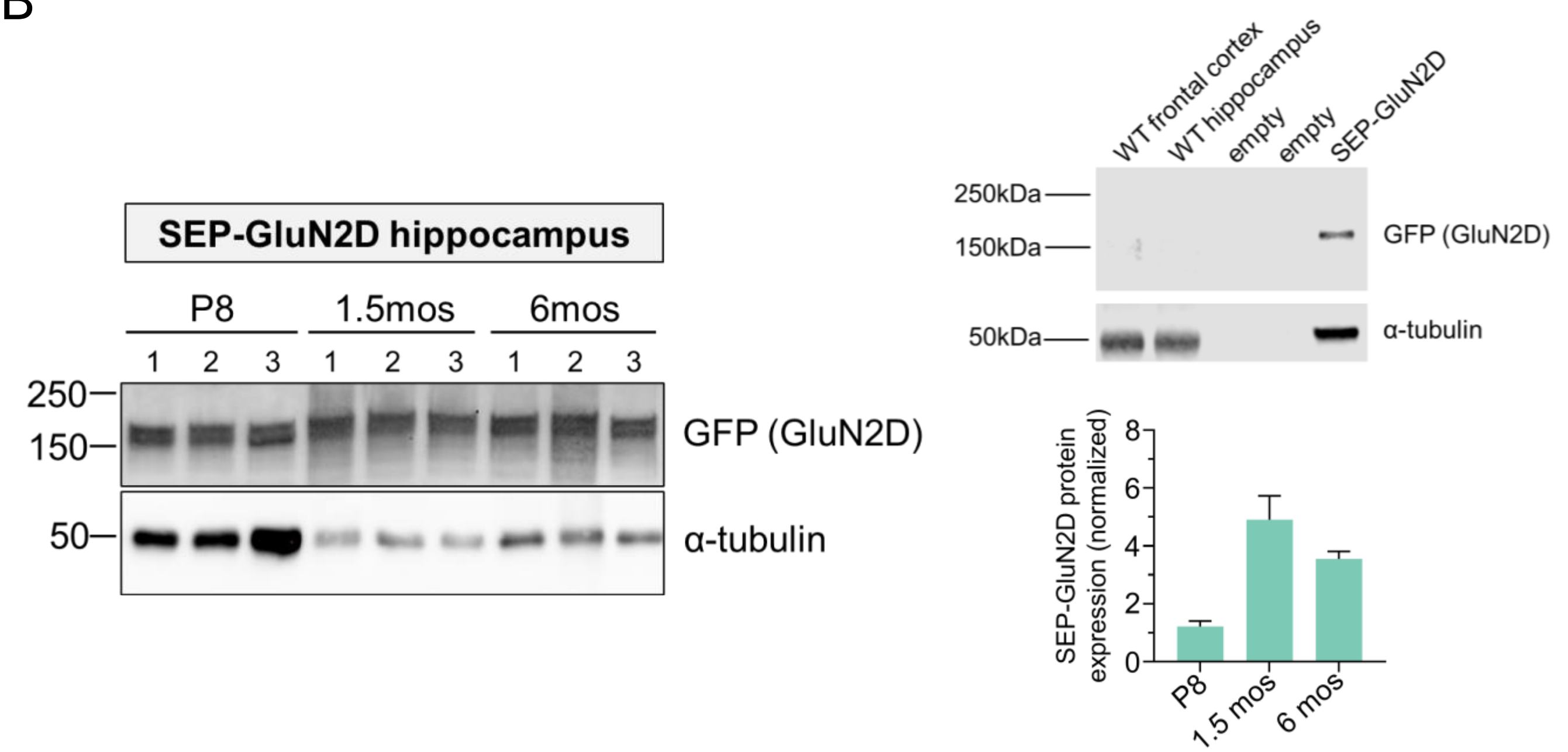

Supplemental Figure S1: Design of *Grin2d* knock-in of SEP into exon 1. **A,** The refseq was (NC_000073.7:45,481,307-45,520,708 reverse strand on Chromosome 7), and the sequencing primer was (GTGCGCCAGCCCGTTCCCGG). The long ssDNA donor (1111bp) is shown for the CRISPR/Cas9 knock-in strategy to insert SEP (magenta) at a position 9 base pairs downstream of the signal peptide (blue). **B,** *left* Western blot of homogenized hippocampal tissue from SEP-*Grin2d* mouse brains at the indicated ages and probed with anti-GFP (to detect SEP-GluN2D) or anti-tubulin antibodies. *Right, top* Western blot validating anti-GFP antibody used to detect SEP-GluN2D. The SEP-GluN2D lane is a positive control sample of HEK293 cells transfected to express SEP-GluN2D protein. *Right, bottom* Quantification of SEP-GluN2D protein expression normalized to tubulin. The MW of the immunoreactive band was consistent with SEP fused to GluN2D (~170 kDa) in both Western blots.

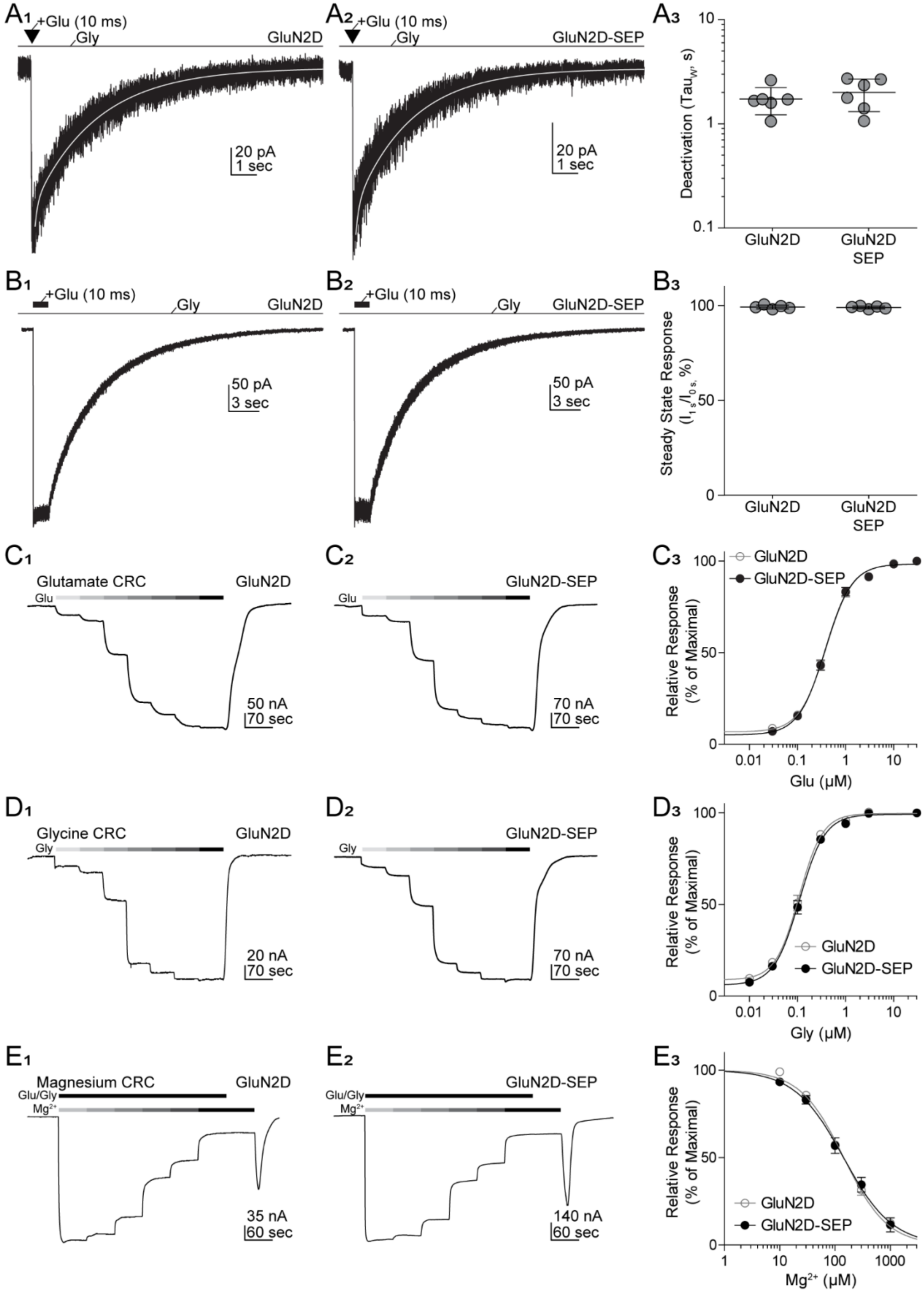

A1
+Glu (10 ms)
Gly
GluN2D
20 pA
1 sec
A2
+Glu (10 ms)
Gly
GluN2D-SEP
20 pA
1 sec
A3
Deactivation (Tau_W, s)
GluN2D
GluN2D SEP
B1
+Glu (10 ms)
Gly
GluN2D
50 pA
3 sec
B2
+Glu (10 ms)
Gly
GluN2D-SEP
50 pA
3 sec
B3
Steady State Response (I1s/I0s, %)
GluN2D
GluN2D SEP
C1
Glutamate CRC
GluN2D
Glu
50 nA
70 sec
C2
GluN2D-SEP
Glu
70 nA
70 sec
C3
GluN2D
GluN2D-SEP
Relative Response (% of Maximal)
Glu (μM)
D1
Glycine CRC
GluN2D
Gly
20 nA
70 sec
D2
GluN2D-SEP
Gly
70 nA
70 sec
D3
Relative Response (% of Maximal)
GluN2D
GluN2D-SEP
Gly (μM)
E1
Magnesium CRC
GluN2D
Glu/Gly
Mg2+
35 nA
60 sec
E2
GluN2D-SEP
Glu/Gly
Mg2+
140 nA
60 sec
E3
Relative Response (% of Maximal)
GluN2D
GluN2D-SEP
Mg2+ (μM)

Supplemental Figure 2: Functional properties of GluN1/GluN2D-SEP NMDA receptors are identical to wild type GluN1/GluN2D receptors. **A**, A representative whole cell current response recorded under voltage clamp (V_{HOLD}, -60 mV) from HEK cells transiently expressing GluN1/GluN2D (**A1**) and GluN1/GluN2D-SEP (**A2**). A summary scatter plot of weighted tau values for all recorded HEK cells expressing either GluN1/GluN2D or GluN1/GluN2D-SEP is shown (**A3**). The mean ± sem fitted tau values for WT GluN1/GluN2D were tau_{FAST} 294 ± 153 ms (37 ± 9.9 %) and tau_{SLOW} 4860 ± 2390 ms. For GluN1/GluN2D-SEP, similar individual tau values were obtained, with a tau_{FAST} of 338 ± 165 ms (37 ± 4.8 %) and a tau_{SLOW} of 3400 ± 640 ms. **B**, A representative whole cell current response recorded under voltage clamp (V_{HOLD}, -60 mV) from HEK cells transiently expressing GluN1/GluN2D (**B1**) and GluN1/GluN2D-SEP (**B2**). A summary scatter plot of steady state response (prolonged glutamate application response/initial glutamate response) for all recorded HEK cells expressing either GluN1/GluN2D or GluN1/GluN2D-SEP is shown (**B3**). The mean ± sem steady state values for WT GluN1/GluN2D were 99 ± 0.44% and GluN1/GluN2D-SEP 99 ± 0.34%. **C**, Representative two-electrode voltage-clamp current recordings showing a glutamate concentration-response curve (CRC) recorded in the presence of 30 µM glycine from *Xenopus laevis* oocytes expressing GluN1/GluN2D (**C1**) and GluN1/GluN2D-SEP (**C2**). Concentration-response curves for glutamate (Glu) were fitted by the Hill equation (**C3**). This panel was shown in Figure 1C, and is included here to allow readers to get a comprehensive picture of the functional properties of GluN2D-SEP. The fitted EC_{50} values for glutamate were 0.39 [0.35,0.43 95% CI] µM (WT) and 0.38 [0.34, 0.42] µM (GluN2D-SEP), determined from log EC_{50}. **D**, Representative two-electrode voltage-clamp current recording of a glycine concentration-response experiment (recorded in the presence of 30 µM glutamate) for oocytes expressing GluN1/GluN2D (**D1**) and GluN1/GluN2D-SEP (**D2**). Concentration-response curves for glycine were fitted with the Hill equation (**D3**); fitted EC_{50} values for glycine were 0.11 [0.09, 0.13] µM (WT) and 0.11 [0.09,0.13] µM (GluN2D-SEP). **E**, Representative two-electrode voltage-clamp current recordings of a Mg^{2+} concentration-response experiment (recorded in the presence of 100 µM glutamate and 30 µM glycine) for oocytes expressing GluN1/GluN2D (**E1**) and GluN1/GluN2D-SEP (**E2**). Concentration-response curves for Mg^{2+} were fitted by the Hill equation (**E3**); the mean fitted IC_{50} value for Mg^{2+} inhibition was 144 [130, 158] µM for WT GluN1/GluN2D and 143 [117, 169] µM for GluN1/GluN2D-SEP. All data are from 8-12 oocytes isolated from two different frogs, with independent experiments completed on two different days.

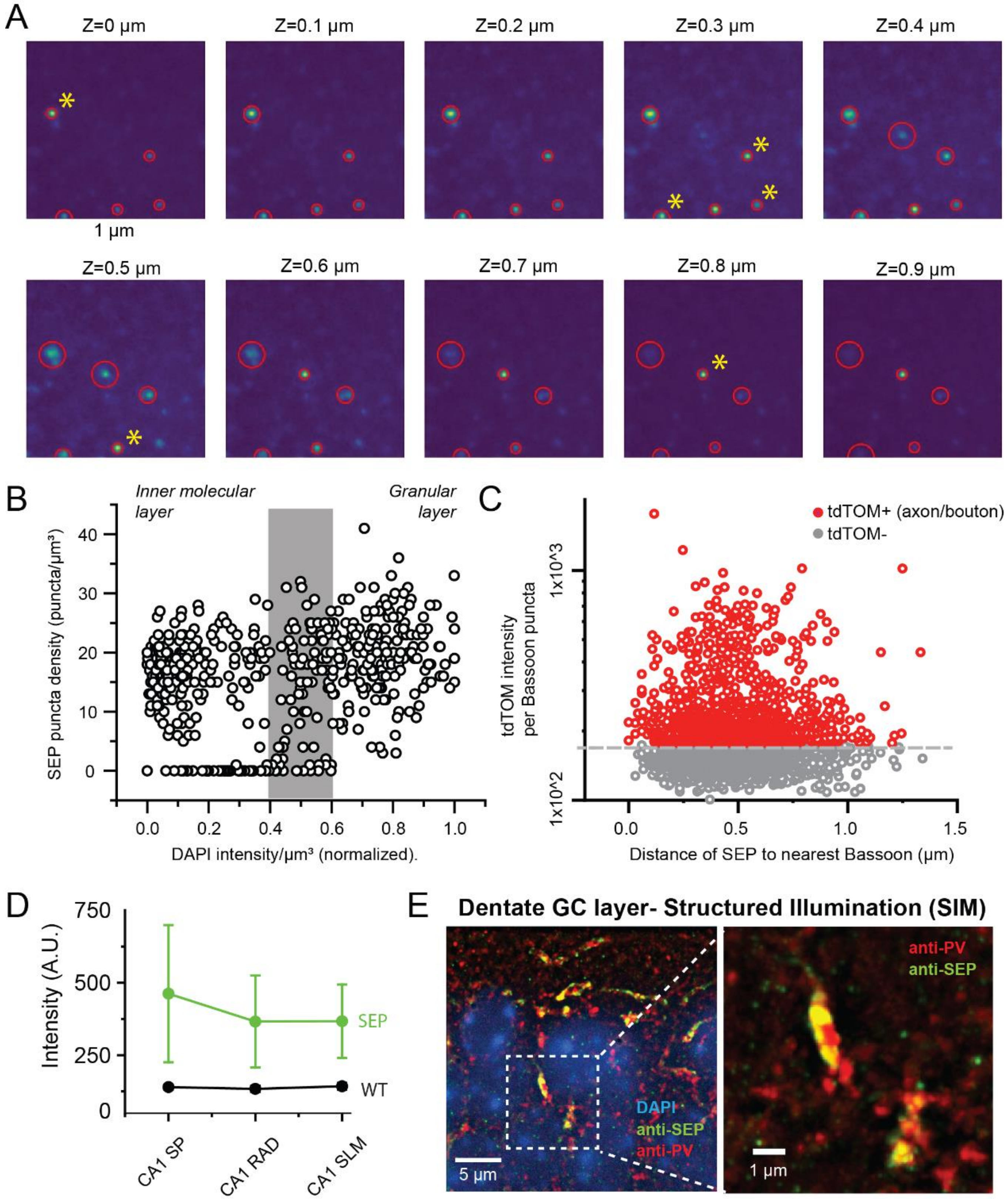

Supplemental Figure S3: SoRa automated SEP puncta analysis and Structured Illumination (SIM) super-resolution microscopy. A, Example images illustrating automated puncta detection within a z-stack collected on the SoRa microscope (step size = 0.1 µm) from an anti-GFP antibody-stained hippocampal section. Yellow asterisks

denote the estimated 3-dimensional center for each identified puncta. **B,** SEP puncta density per μm^3 from z-stack image stacks, including the inner molecular (IML) and granule cell (GL) layers. IML and GL were designated based on the corresponding DAPI signal intensity. Note that statistical analysis (e.g., ANOVAs in main **Figure 1G**) of SEP density for IML and GL omit data from within the IML-DG transition zone (gray box). **C**, Bassoon and SEP puncta nearest neighbor distance quantified on three granule cell layer z-stacks from three GluN2D-SEP animals. Red circles denote the bassoon puncta that were located within tdTom-labeled boutons/axons, as a function of distance from the nearest SEP puncta. **D,** Integrated total absolute fluorescence intensity of green signal from the CA1 SP, RAD, and SLM regions across SEP (n=3) and WT (n=2) mice. **E,** Independent experiment from an anti-GFP and anti-PV co-stained slice (z-stack depth 10 μm) imaged at the IML-granule cell layer border in the dentate on a Structured Illumination Microscope (SIM). Images for red, green, and blue fluorescence are superimposed. DAPI-label GCs were surrounded by PV+ putative basket synapse swellings. Enlarged view (*right*) from the boxed inset shows a PV+ putative basket bouton with substantial anti-SEP co-labeling.

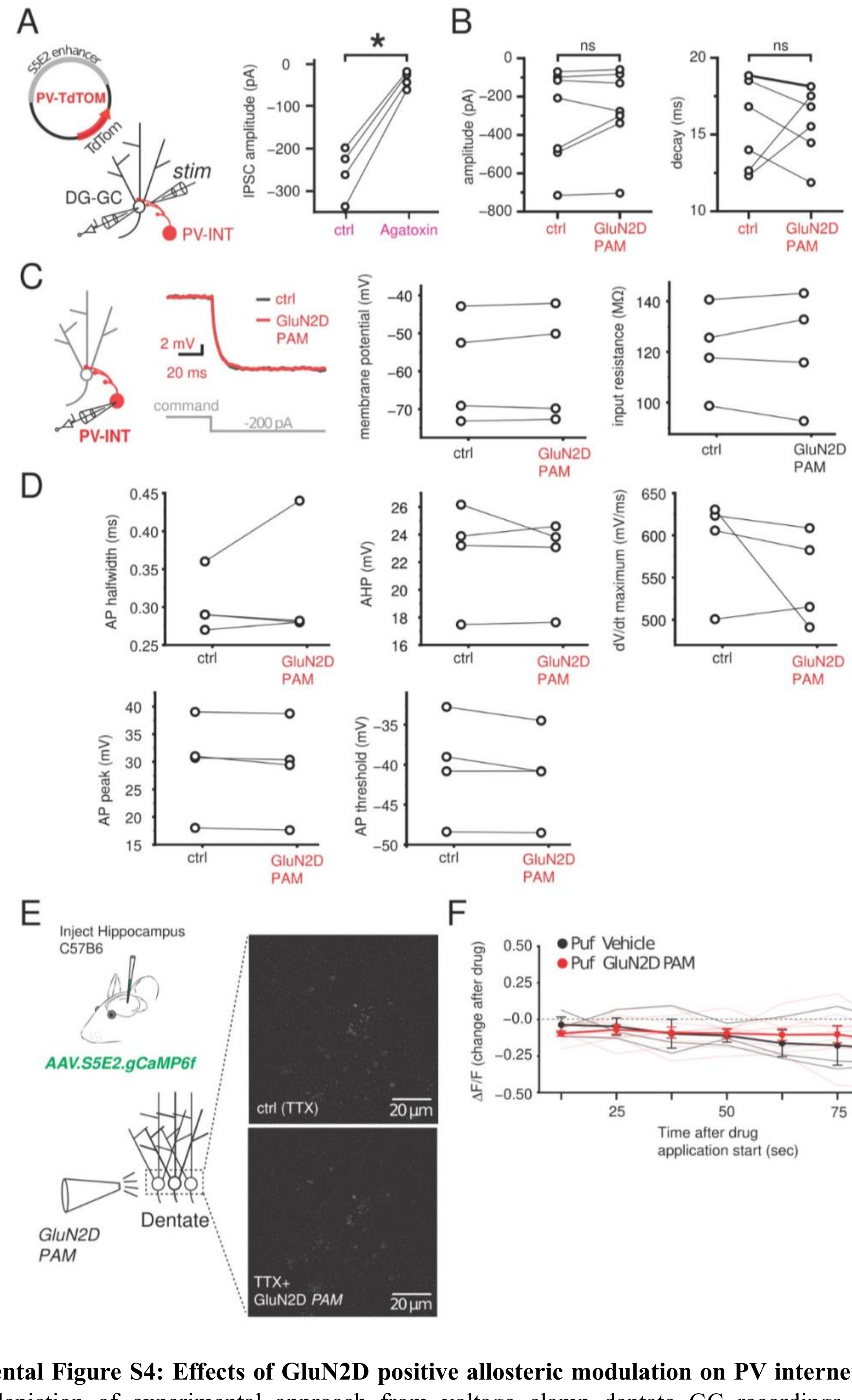

Supplemental Figure S4: Effects of GluN2D positive allosteric modulation on PV interneuron properties. A, Cartoon depiction of experimental approach from voltage clamp dentate GC recordings coupled with local stimulation. On the *left*, Agatoxin (0.5 µM) greatly reduces stimulus-evoked IPSCs (paired t-test, n = 4). **B,** IPSC amplitudes and kinetics were unaltered by GluN2D-selective PAM application (paired t-test, n=7). **C,** Current clamp

recordings of fast spiking PV interneurons in dentate gyrus. The effect of 10 μM (+)EU1180-453 (GluN2D PAM) bath application on PV passive features is summarized. **D,** Effect of (+)EU1180-453 bath application on PV interneuron action potential and active properties are summarized. All data in (**C**) and (**D**) were non-significant by paired t-tests. **E,** Adult WT mice were injected with AAV.S5E2.gCaMP6f in the hippocampus. 2 weeks later, stable punctate basal Ca^{2+} signals were detectable with TTX in the bath in the expected peri-somatic pattern of basket terminals. After averaging basal signals in TTX over several minutes, (+)EU1180-453 was continuously, locally puffed using a second pipette adjacent to the FOV. We ensured that the drug washed over our area of interest by including a red (Alexa 594) dye in the puff pipette to visualize drug application during the experiment. We used stringent criteria to ensure movement artifacts did not alter signal collection for individual puncta during local drug application (see Methods). **F,** Summary data of changes over time for all puncta imaged after PAM (n= 389 boutons from 9 slices) showed virtually no changes in Ca^{2+} signal over time.

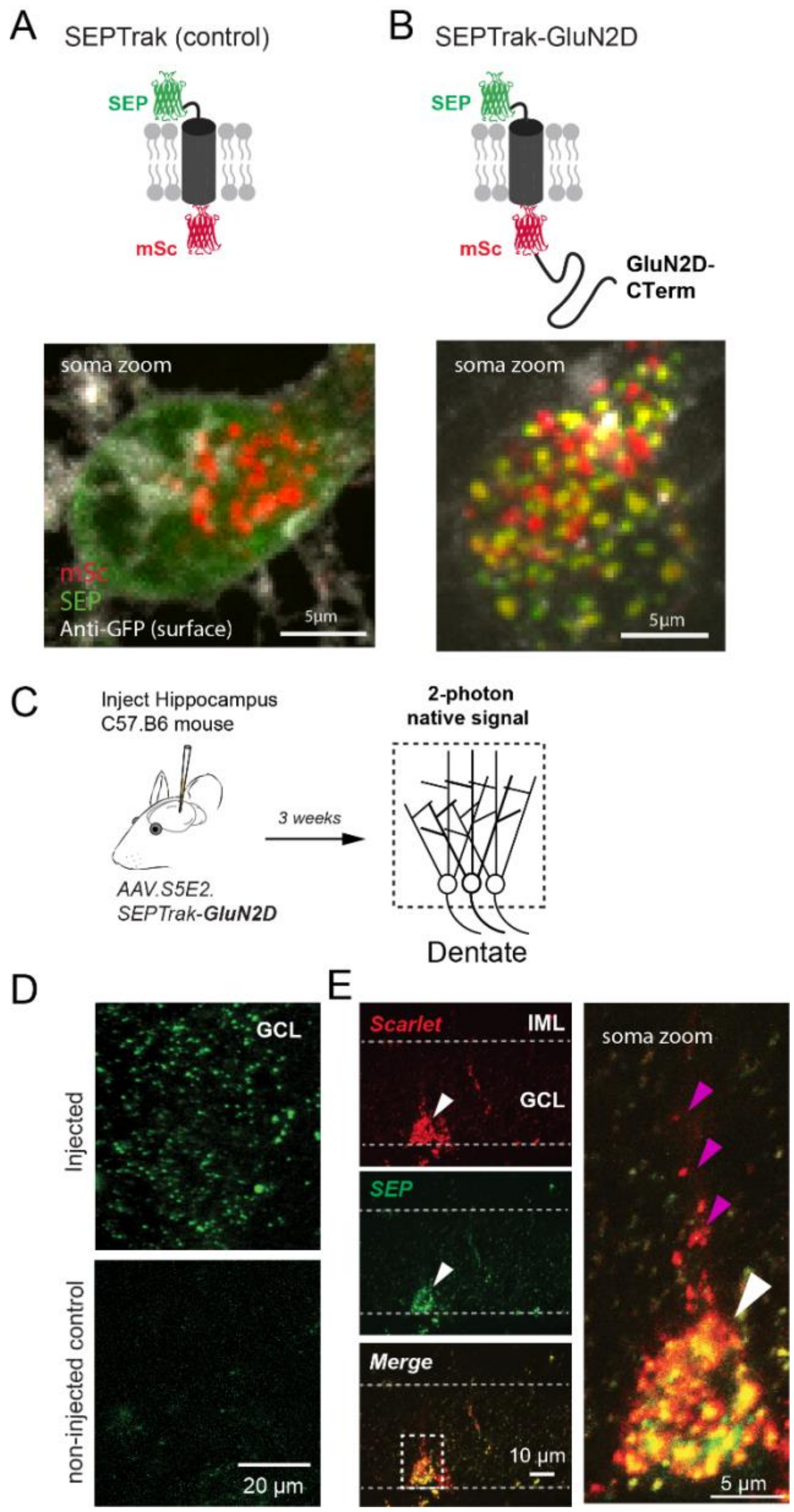

Supplemental Figure S5: GluN2D C-terminus expression is visible in intracellular somatic structures. Cultured hippocampal neurons were transfected at DIV17 with either SEPTrak **A**, or SEPTrak-GluN2D **B**, and imaged 2 days later following 10-15 min of surface labeling with an extracellular anti-GFP nanobody (greyscale signal). Intracellular somatic labeling is apparent. **C,** Cartoon illustrating *in vivo* administration of AAV delivering S5E2-driven SEPTrak-GluN2D to hippocampal parenchyma. **D,** 2-photon imaging of SEP signal in the GCL from injected and non-injected animals, imaged using identical parameters on the same day. **E,** mScarlet (mSc), SEP, and merged images from a PV neuron soma (white arrow) in the dentate region, with a zoomed-in view of the soma and putative proximal dendrite (magenta arrows) shown. Note the similarity of the intracellular somatic signal in vivo (*this panel*) and in vitro (*panel B*). Although sparsely SEPTrak-GluN2D-labeled, when seen, putative PV proximal dendrites showed red-shifted intracellular signals (*as in this panel*).

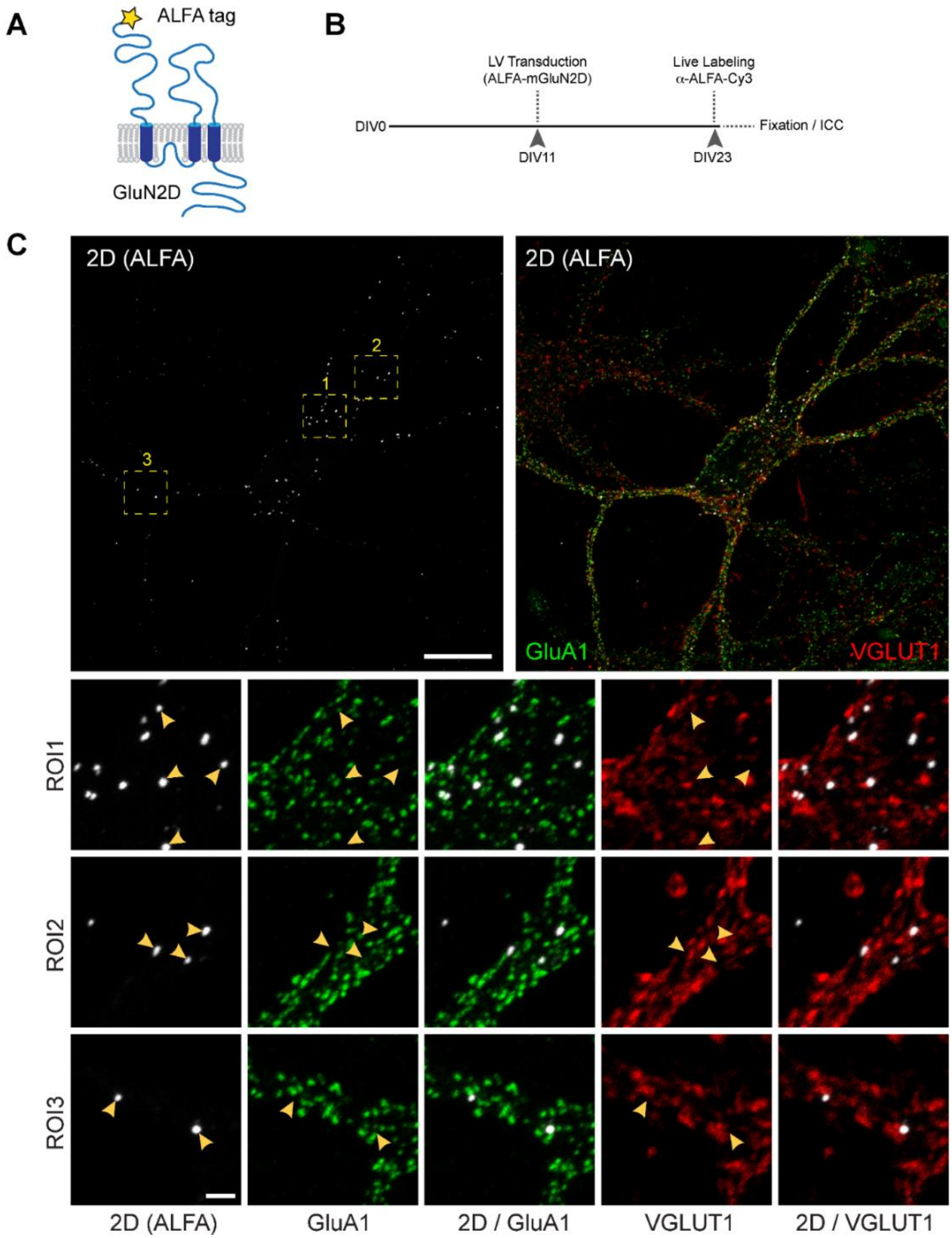

Supplemental Figure S6: Subcellular localization of full-length ALFA-tagged GluN2D. Rat primary cultured hippocampal neurons were transduced at DIV11 with a lentivirus expressing full-length mouse GluN2D tagged with the ALFA epitope near the extracellular N-terminus. Neurons were live-labeled on DIV23 with Cy3-conjugated anti-ALFA and anti-GluA1 (extracellular), fixed, and labeled with anti-VGLUT1. **A,** Schematic of ALFA-tagged GluN2D,

B, experimental strategy, and **C,** expanded view of a representative erbB4+ positive interneuron with three regions of interest (ROIs) outlined by bounding boxes. Surface ALFA-GluN2D is shown on the *left*, the merged image on the *right*. Magnified views showing the ROIs, including separate channels for ALFA-GluN2D, GluA1, and VGLUT1, as well as merged images for GluN2D/GluA1 and GluN2D/VGLUT1. Arrowheads indicating the location of some ALFA-GluN2D puncta illustrate the lack of corresponding GluA1 and VGLUT1 signals. Data are representative of five independent experiments. Scale bars = 20 µm (overviews), 2 µm (ROIs).

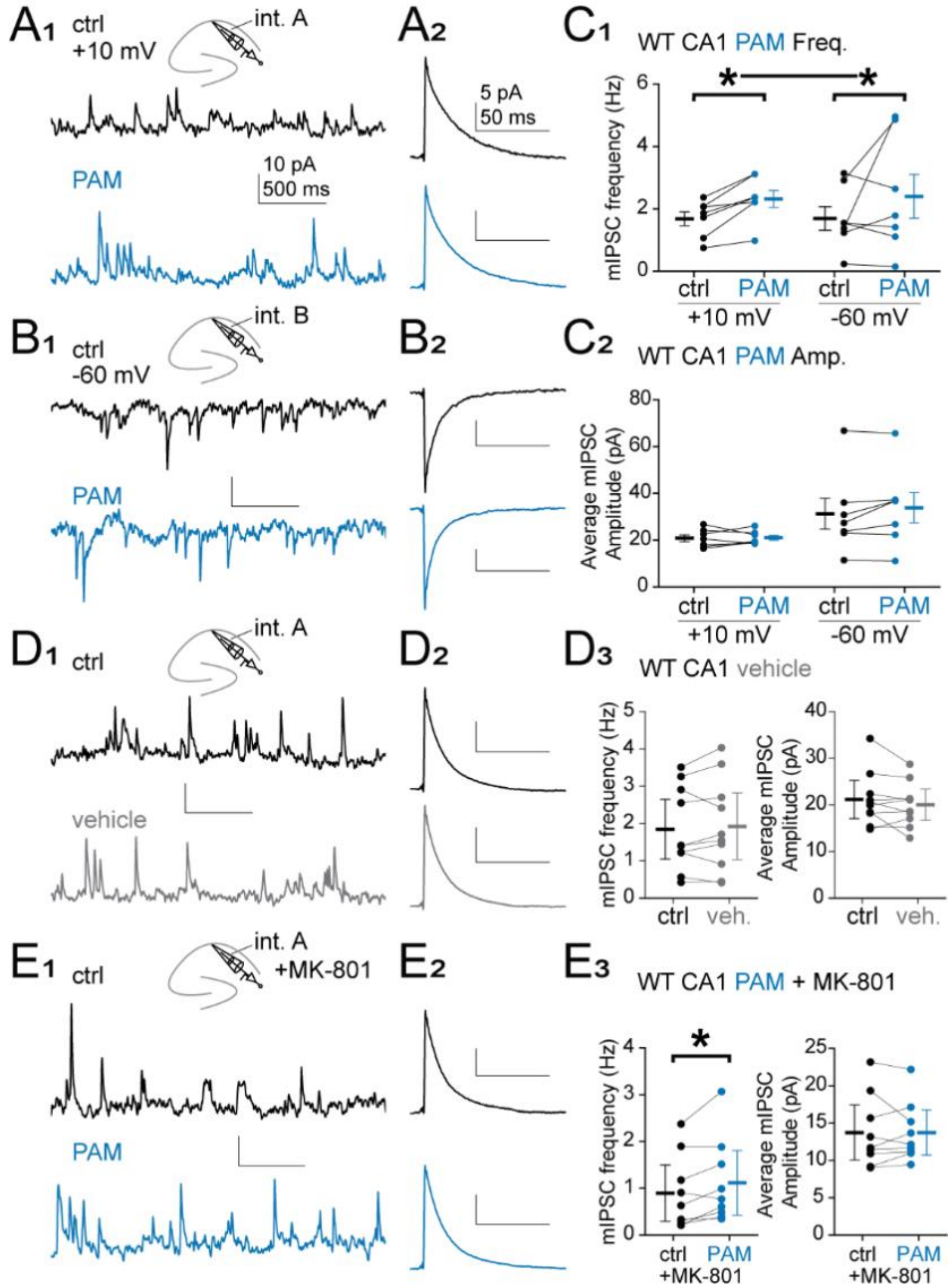

Supplemental Figure S7: Postsynaptic internal recording solution does not alter the effects of GluN2D modulation of mIPSC frequency. A1, Whole cell patch clamp recording from a CA1 pyramidal cell in 1 μM TTX held near the AMPA receptor reversal potential (+10 mV). Outward currents are mIPSCs. **A2**, Average time course of mIPSCs. **B1**, Whole cell current recording from a CA1 pyramidal cell held at –60 mV in the presence of the competitive AMPA receptor antagonist 10 μM CNQX. The holding potential and internal solution (see Methods) rendered mIPSCs as inward currents. **B2**, Average mIPSC time course. **C1**, There was a significant effect by 2-way ANOVA whereby the drug was different than baseline for each respective recording condition. **C2**, There was no significant difference in the degree of potentiation between different holding potentials; 2-way ANOVA, significant effect with PAM ($F_{(1,16)} = 7.93$, p=0.0124), no interaction ($F_{(1,16)} = 1.08$, p=0.3145) or internal solution ($F_{(1,16)} = 0.04$, p=0.8509) effect. **D**, A representative recording is shown for the full duration of an experiment in vehicle (**D1**), demonstrating that recordings were stable with no drift in mIPSC composite waveform (**D2**) frequency or amplitude (**D3**). **E**, Inclusion of MK801 in the pipette solution did not alter the response of mIPSCs to GluN2D modulation, displayed similarly to **D**. Data are mean (large hash) ± STD (small hash). * indicates p<0.05 (paired t-test). For frequency, WT vehicle p=0.46 and WT MK801+PAM p=0.014; for amplitude, WT vehicle p=0.21 and WT MK801+PAM p=0.62. See **Supplemental Tables S1,S2** for all statistical parameters.

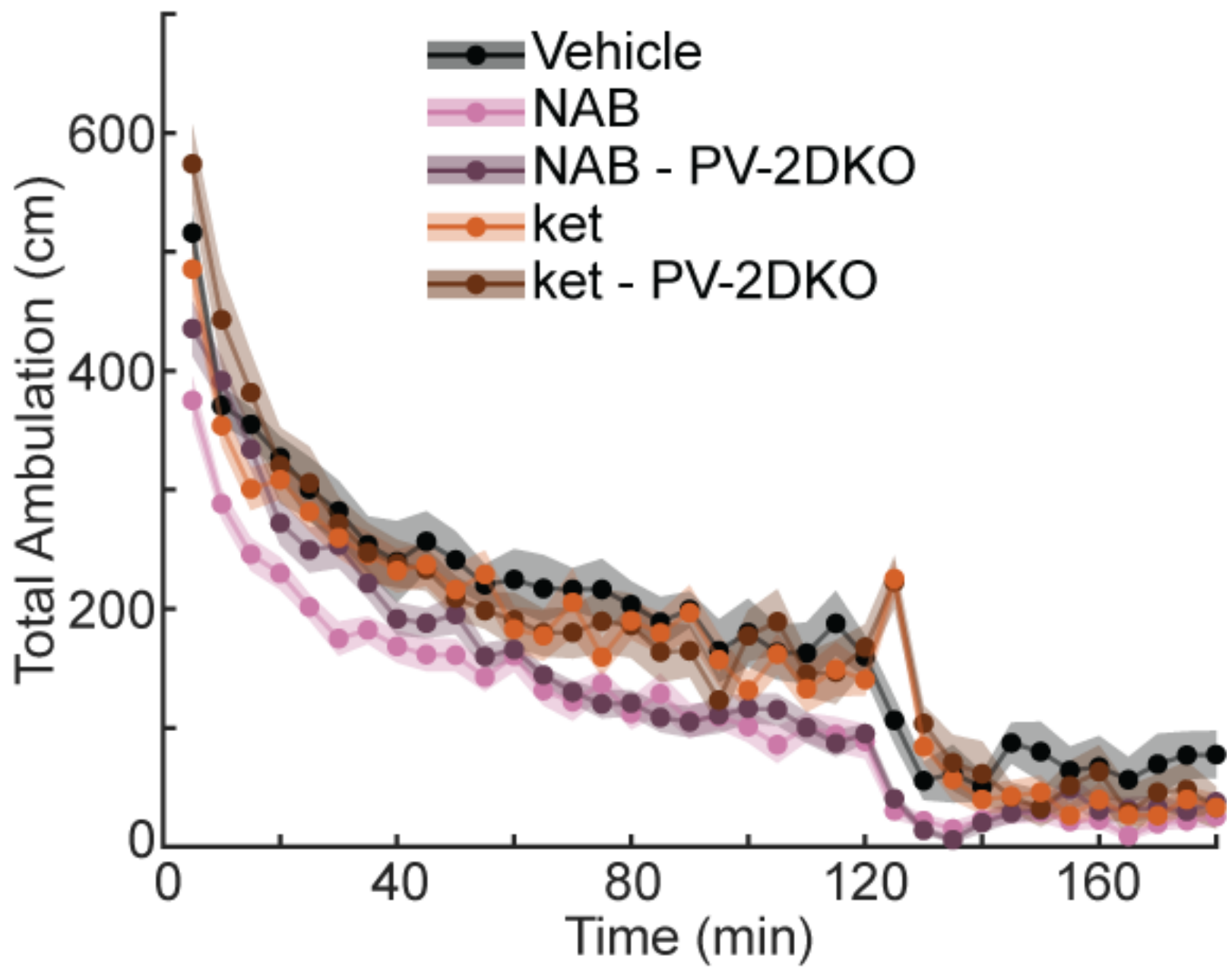

Supplemental Figure S8. Time course of locomotor response to NAB14, ketamine, and vehicle. The time course of ambulation in 5 min time bins is shown for adult male and female mice injected with 50% PEG / 10% DMSO in sterile saline vehicle alone, and with 10 mg/kg (10 ml/kg dose volume of 1 mg/ml solution) of either ketamine, NAB14, or vehicle.
